# Measuring and evaluating participant understanding of consent processes in clinical trials: a systematic review

**DOI:** 10.1186/s13063-026-09582-x

**Published:** 2026-03-04

**Authors:** Saba Faisal, Julia Wade, Jhulia dos Santos, J. Athene Lane, Giles Birchley, Sarah Dawson, Shoba Dawson

**Affiliations:** 1 Bristol Medical School, University of Bristol, Bristol, UK; 2 London School of Hygiene & Tropical Medicine, London, UK; 3 School of Medicine and Population Health, University of Sheffield, Sheffield, UK

**Keywords:** Clinical trials, Consent process, Recruitment discussion, Participant understanding, Tools/measures

## Abstract

**Background:**

Informed consent (IC) is essential for maintaining participant autonomy in clinical trials by ensuring participants are fully informed. However, inconsistent oversight of spoken information provision and participant comprehension of both written and spoken information can lead to significant gaps in participant understanding and recall of critical trial details. This systematic review (SR) evaluates existing tools or approaches that measure participant understanding during the IC process. It will further focus on the quality of data regarding the validity and reliability of these methods.

**Methods:**

Relevant primary studies were identified through searching electronic databases from inception to March 2023. Studies included adults who had undergone the IC process for research. Following screening, data extraction was performed using a customised Microsoft Excel template, focusing on characteristics including validity, reliability, and patient and public involvement in the development of tools/measures used to assess participant understanding. Narrative synthesis was used to descriptively organise and summarise findings across studies, including study characteristics, assessment timing, and types of tools or approaches used, while psychometric properties were evaluated using the COSMIN (COnsensus-based Standards for the selection of health Measurement INstruments) framework.

**Results:**

Of the 6526 records screened, 261 studies were retrieved for full-text screening and a total of 148 studies were included in the review. Among these studies, 103 were quantitative, 24 were mixed methods, and 20 were qualitative studies. This SR identified variability across tools/measures and approaches used in clinical trials to measure participant understanding of IC. Only three tools demonstrated high-quality psychometric properties, i.e. the Digitised Informed Consent Comprehension Questionnaire (DICCQ), the Participatory and Informed Consent (PIC) tool, and the Process and Quality of Informed Consent (P-QIC). Notably, the most frequently used tool across studies, the Quality of Informed Consent (QuIC) questionnaire, demonstrated relatively low methodological quality in its reported psychometric properties. In addition, patient and public involvement in the development of these tools was infrequently reported and often limited in scope.

**Conclusions:**

This review highlights a disconnect between psychometric rigour and common practice. It also emphasises the need to strengthen the validation and standardisation of assessment approaches, alongside more consistent and meaningful integration of patient and public perspectives in their development and validation.

**Registration:**

PROSPERO ID: CRD42023407715. Version 1.1, published 14 Aug 2025. Version 1.0, published 22 Mar 2023

**Supplementary Information:**

The online version contains supplementary material available at 10.1186/s13063-026-09582-x.

## Background

Clinical trials are fundamental to advancing medical knowledge and improving patient outcomes [1], and informed consent (IC) is ethically and legally essential for ensuring participant autonomy and safety when participating in clinical trials [2, 3]. IC implies that participants make informed decisions regarding their participation on their own accord; therefore, ensuring that their rights are protected and ethical standards are met [4].

International guidelines stipulate that IC must be based on fully understanding the critical trial information provided during the recruitment discussions [5–7]. In this review, critical trial details refer to core elements commonly required for valid informed consent, including the study purpose, procedures, risks and potential benefits, randomisation, voluntariness, alternatives to participation, and the right to withdraw, consistent with international ethical guidance [5–7]. This information may not be adequately understood, raising concerns regarding the adequacy of IC in practice [8–11]. This is more prevalent in clinical trials, where the complexity of information creates a huge burden for prospective participants [12]. The absence of standardised tools and methods for assessing participant comprehension further complicates the problem [9]. The heterogeneity of the IC process across different studies and healthcare settings underscores the need for standardised measures to evaluate patient understanding and drive the improvement in the quality of information provision in clinical trials [13, 14].

Despite the existence of several non-standardised tools/measures aimed at assessing understanding for IC processes, systematic reviews (SR) evaluating these tools and their characteristics are lacking [10, 15]. In existing literature, there are reviews of some instruments [16, 17], for example, the Quality of Informed Consent (QuIC) measure [18] and the Brief Informed Consent Evaluation Protocol (BICEP) [19], but very little is known regarding their reliability and validity.

The principal questions guiding this systematic review are as follows:

1. What tools/measures and approaches exist for evaluating and measuring participant understanding for IC in clinical trials?
2. To what extent have these tools/measures and approaches been validated for their reliability and effectiveness in measuring comprehension?

This SR addresses this research gap by identifying and evaluating existing tools/measures and approaches for measuring participants’ understanding of IC in clinical trials. It examines how participant understanding of informed consent has been assessed in clinical trials, focusing on the characteristics, development, and validation of tools and approaches used for this purpose.

## Methods

This review was conducted and reported by following the guidelines in the Preferred Reporting Items for Systematic Reviews and Meta-Analyses (PRISMA) checklist [20].

### Search strategy

The search strategy employed used a combination of Medical Subject Headings (MeSH) and free-text terms for three concepts, namely ‘Participant’, ‘Informed Consent’, and ‘Understanding’. To maximise the efficiency and precision of the search strategy, it was developed with an information specialist (SaD) and a series of preliminary searches were performed across various electronic databases. These preliminary searches were designed to fine-tune the approach, ensuring it yielded comprehensive yet highly relevant results [21]. Electronic bibliometric medical databases such as MEDLINE (see Supplementary Table 1 for example search strategy), EMBASE, PsycINFO, and Cumulative Index to Nursing and Allied Health Literature (CINAHL) were searched from inception until March 2023. In addition to the database searches, the search was supplemented by conducting forward and backward citation searches [22].

### Inclusion and exclusion criteria

The inclusion and exclusion criteria for this review encompassed the following conditions, leading to the selection of relevant studies:

*Population*: Studies which recruited adults aged 18 years or over, undergoing IC for research. Research that documented IC processes for children, adults lacking decision-making capacity, or consent provided by surrogates was excluded. Studies focused on IC for clinical care medical procedures outside a research context were excluded.

*Intervention*: The included studies were required to have reported on tools/measures or approaches that measured understanding of IC (or its aspects) in research studies. The studies were eligible for inclusion if they reported: (i) assessment approaches incorporating items or domains derived from previously validated tools/measures or approaches that measured participant understanding of IC; (ii) the development or validation of a new tool/measure or approach; or (iii) activities related to scale development and validation. The inclusion criteria included any approach that was self-reported, administered by interview, or conducted through proxy. In addition, there was no comparator or control.

*Settings/context*: The inclusion criteria for this review encompassed any setting in which a tool/measure or approach was used to assess patient understanding of recruitment discussions and information required for participation in a clinical trial.

*Main outcomes*: The outcome of participant understanding was categorised into immediate knowledge (tested within 1 week of the control or intervention procedure) and knowledge retention (no time limit).

*Language*: The search strategy was tailored to encompass only English-language articles.

*Study types*: Primary studies of any design (quantitative, qualitative, and mixed methods) were included. Theses were also included, whereas conference abstracts, editorials, books/book chapters, letters/commentaries, other forms of grey literature, and SRs were excluded. The reference lists of relevant SRs were scanned to identify other studies for potential inclusion.

### Study selection

EndNote software (version 20) was used to combine and export the search results obtained from various databases. Prior to the study selection process, a total of 6526 records were obtained from database searches. A total of 1736 duplicate records were removed using the EndNote software, and 4790 records were screened on Rayyan software [23]. The study selection was conducted in two stages. Firstly, two reviewers (SF and JdS) independently screened all titles and abstracts to identify eligible and relevant studies. The inter-rater reliability between the two reviewers was calculated as 0.87, indicating a high level of agreement and consistency in their decisions regarding article inclusion or exclusion.

In addition, the kappa coefficient between the two reviewers was calculated using the formula Kappa = (Po − Pe)/1 − Pe. The kappa coefficient between the two reviewers (SF and JS) was calculated to be 0.79, further indicating a high level of agreement that considers the possibility of chance agreement. There were 60 articles with a conflicting decision; these were resolved through discussions facilitated by two reviewers (JW and SD) until consensus was reached.

**Fig. 1:**
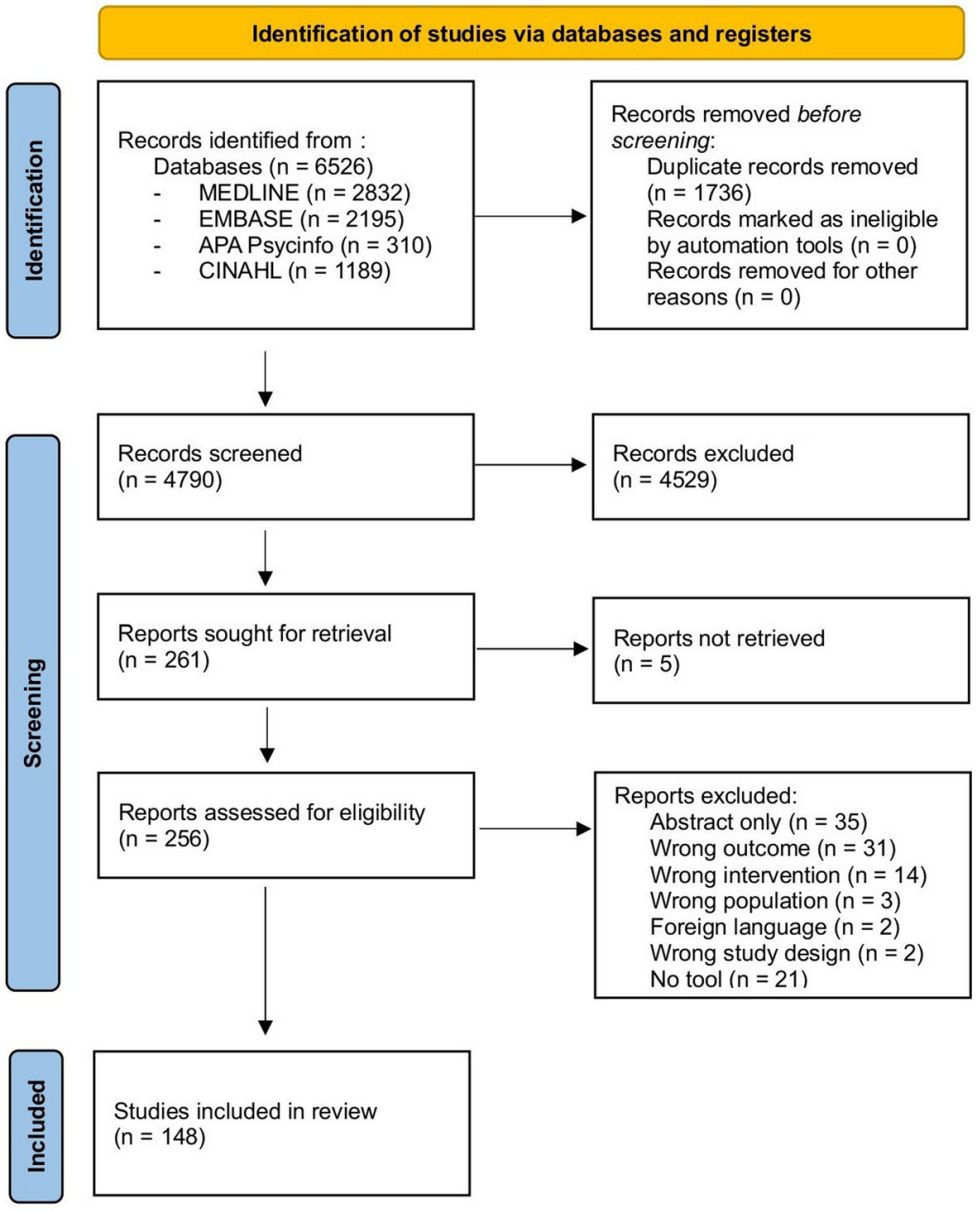
PRISMA flow diagram

A total of 256 articles were taken to the next stage and full texts were retrieved. Of these, 25 studies lacked sufficient detail on the tools/measures and approaches used. Therefore, we contacted the corresponding author (where possible) to request for it. Four authors responded and provided the requested material. These full-text articles then underwent the second screening process based on the same eligibility criteria by two independent reviewers (SF and JdS), with conflicts resolved by JW and SD. Finally, a total of 148 papers were included in the review. The detailed PRISMA flow diagram is shown in Fig. 1.

### Data extraction and critical assessment

A customised Microsoft Excel document was used to extract the details from the full texts which included the following tool/measure characteristics: name of the tool/measure; constructs and domains being targeted by item (verbatim from included study); time required to complete; recall period; validity of the tool; reliability of the tool; and Patient and Public Involvement and Engagement (PPIE) involvement at development stage. Psychometric properties were extracted directly from the included studies.

The data extraction form was pilot tested on a random sample of 10% of included studies. This allowed us to refine the data extraction form as required [24]. One reviewer (SF) extracted the data for consistency, which was double-checked by a second reviewer (JW). The discrepancies were resolved through discussion or the involvement of a third reviewer. Data extraction was performed by one reviewer (SF), and 30% of the data was independently verified collectively by JW, SD, AL, and GB to ensure accuracy.

Risk of bias (quality) assessment was not conducted, as it focused on identifying measures that assess patient understanding for IC when participating in research. The quality of the studies was not used as a determining factor in their selection or in the synthesis of results [25]. However, these tools/measures were further analysed by applying COSMIN (COnsensus-based Standards for the selection of health Measurement INstruments) framework which is an approach to assess psychometric properties such as reliability and validity aspects in a standardised manner [26].

### Data synthesis

Data were synthesised using the guidance provided by Popay’s framework on narrative synthesis, specifically focusing on elements 2 and 3 [27]. These elements entail developing an initial portrayal of the outcomes and exploring relationships within and between studies. By adhering to this framework, the studies included in the research were examined, and data synthesis was structured. Data were synthesised using narrative synthesis to provide a structured descriptive summary of included studies, focusing on study characteristics, timing of assessment, and the types of tools/measures or approaches used to assess participant understanding. Where studies reported modified or translated versions of existing tools, these were treated as separate tools when psychometric testing of the adaptation was reported; otherwise, psychometric evaluation was based on the original version of the tool.

## Results

### Study demographics

Table 1 demonstrates characteristics of all 148 studies included [11, 18, 28–173] and their participants.

**Table 1:** Characteristics of the study and the participants

#	Author year	Country	Study design	Population	PRO-EDI domains reported	Construct assessed
1	Abd-Elsayed et al. 2012 [28]	USA	Quantitative	Patients eligible for trials (delit, glucose, blood age)	Age; sex/gender; race/ethnicity; education level	Communication; satisfaction
2	Addissie et al. 2016 [29]	Ethiopia	Quantitative	Pregnant women between 18 and 45 years attending ANC follow-up in four selected health facilities and who were targeted for the HPV sero-prevalence study	Age; marital status; race/ethnicity; religion; language; education level; occupation; income; previous clinical trial participation	Quality of IC process; comprehension; recollection
3	Afolabi et al. 2014 [30]	The Gambia	Quantitative	Among participants in two clinical trials	Age; sex/gender; location; occupation; education level; religion; previous clinical trial participation	Comprehension
4	Afolabi et al. 2015 [31]	The Gambia	Quantitative	Participants in a malaria treatment trial in Basse and Jahaly Provinces	Age; sex/gender; location; race/ethnicity; education level; religion; previous clinical trial participation	Comprehension
5	Ahalt et al. 2017 [32]	USA	Quantitative	Incarcerated older adults	Age	Comprehension
6	Alexa-Stratulat et al. 2018 [33]	Romania	Quantitative	Clinical trial participants (50 were healthy volunteers participating in phase I clinical trials and 50 were cancer patients participating in phase II and III clinical trials)	Age; sex/gender; location; education level; previous clinical trial participation	Understanding
7	Allen et al. 2017 [34]	USA	Quantitative	Participants in an ongoing RCT (The XR-NTX Relapse Prevention Effectiveness Study)	Age; sex/gender; race/ethnicity; education level; employment status; insurance status	Knowledge
8	Apseloff et al. 2013 [35]	USA	Quantitative	Thirty healthy subjects (age 21–45 years) in a long-duration clinical trial	Age; sex/gender; race/ethnicity; education level; income; employment status; previous clinical trial participation	Comprehension
9	Arora et al. 2011 [36]	India	Quantitative	Healthy male volunteers participating in a phase I, first-in-human study	Age; sex/gender; education level; employment status	Comprehension
10	Asher et al. 2022 [37]	Israel	Quantitative	Patients’ currently receiving active anticancer therapy at a large tertiary hospital	Age; sex/gender; country of birth; religion; education level; income; location; previous clinical trial participation	Understanding; attitude
11	Atal et al. 2018 [38]	Ireland	Mixed methods	Pregnant women diagnosed with gestational diabetes mellitus (GDM) enrolled in the EMERGE clinical trial	Age; sex/gender; race/ethnicity; education level	Comprehension
12	Ballard et al. [39]	UK	Mixed methods	Participants of the 100,000 Genomes Project, including patients with rare diseases, cancer patients, and their family members	Age; sex/gender; education level	Recollection
13	Barrett 2005 [40]	USA	Quantitative	Patients who recently consented to enrol in oncology clinical trials	Age; sex/gender; race/ethnicity; education level	Knowledge; understanding
14	Behrendt et al. 2011 [41]	Germany	Qualitative	Cancer patients who were asked to participate in a randomised trial	Age; sex/gender; nationality	Understanding
15	Benson et al. 1985 [42]	USA	Mixed methods	The participants of two studies (the depression study and the schizophrenia study)	Age; sex/gender; race/ethnicity; education level	Understanding
16	Beranger et al. 2019 [43]	France	Quantitative	Parents and children who have been asked to give consent for participation in an early-phase oncology trial	Age; sex/gender; previous clinical trial participation	Understanding
18	Bergenmar et al. 2011 [44]	Sweden	Quantitative	Cancer patients who consented to participate in phase II or III clinical trials at the Department of Oncology, Karolinska University Hospital	Age; sex/gender; marital status; education level; previous clinical trial participation	Knowledge; understanding
17	Bergenmar et al. 2008 [45]	Sweden	Quantitative	Patients who had been informed in Swedish about a phase II or a phase III clinical trial at the Department of Oncology, Karolinska University Hospital, and had signed a consent	Age; sex/gender; education level; marital status; language; previous clinical trial participation	Knowledge; understanding
19	Bhansali et al. 2009 [46]	India	Quantitative	Patients with dyslipidaemia who were invited to participate in a phase 3 multicentric trial of a novel lipid lowering agent	Age; sex/gender; education level; income; previous clinical trial participation	Comprehension
20	Boyd et al. 2021 [47]	New Zealand	Qualitative	Caregivers of children participating in a dental randomised control trial (RCT)	Age; sex/gender; race/ethnicity; location	Understanding
21	Brandberg et al. 2016 [48]	Sweden	Quantitative	Cancer patients planned for information about a cancer clinical trial in phases 2 or 3	Age; sex/gender; education level; marital status; previous clinical trial participation	Knowledge; understanding
22	Campbell et al. 2008 [49]	Mexico	Quantitative	Individuals recruited from outpatient clinic waiting areas at the New Mexico Veterans’ Affairs Health Care System (NMVAHCS) and the University of New Mexico Hospital (UNMH)	Age; sex/gender; race/ethnicity; previous clinical trial participation	Impact
23	Campbell et al. 2017 [50]	South Africa	Quantitative	Participants recruited for cases and controls for the Genomics of Schizophrenia in South African Xhosa People (SAX) project	Age group; sex/gender; education level; location	Understanding
24	Cervo et al. 2013 [51]	Italy	Quantitative	Cancer patients admitted to the Centro di Riferimento Oncologico IRCCS Aviano, who agreed to contribute to its biobank	Age; sex/gender; nationality; education level; marital status	Understanding
25	Chaisson et al. 2011 [52]	Botswana	Quantitative	HIV-infected adults participating in a 3-year clinical trial for tuberculosis prevention	Age; sex/gender; education level; employment status; language of administration	Comprehension
26	Chapman et al. 2015 [53]	Spain, Uganda, USA, and others	Mixed methods	Participants of a multicentre tuberculosis treatment trial	Age; sex/gender; race/ethnicity; country of birth; education level	Comprehension; satisfaction
27	Chappuy et al 2010 [54]	France	Mixed methods	Parents whose consent was sought for their child to participate in the FRALLE 2000 A protocol (acute lymphoblastic leukaemia) at two centres	Age; sex/gender; marital status; language; socio-professional status	Understanding
28	Choi et al 2019 [11]	South Korea	Qualitative	Participants or legally authorised representatives (mostly patients’ close family), who had experienced at least once consent process in anticancer drug clinical trials	Age; sex/gender; education level	Understanding
29	Cohn 2009 [55]	USA	Mixed methods	Health professions students whose curriculum included issues of informed consent for research	Education level; occupation	Quality of IC process
30	Collins et al. 2023 [56]	Canada	Quantitative	Patients at the time they attended a follow-up neurovascular clinic 4 to 52 weeks after a care episode where they did or did not participate in a clinical trial	Age; sex/gender	Understanding
31	Corneli et al. 2012 [57]	Malawi	Quantitative	Pregnant women with unknown HIV status	Age; education level	Understanding
32	Criscione et al. 2003 [58]	USA	Quantitative	Respondents were participants in a 16-week randomised, single site, double-masked, placebo-controlled trial designed to evaluate intravenous doxycycline as a therapy for RA	Age; sex/gender; race/ethnicity; marital status; education level	Understanding
33	da Fonseca et al. 1999 [59]	Brazil	Qualitative	Men who have sex with men, who are HIV negative, and who are between 18 and 49 years old	Education level; race/ethnicity; marital status; employment status	Knowledge
34	Das et al. 2014 [60]	Bangladesh	Qualitative	Adult clinical trial participants with uncomplicated falciparum malaria	Age; sex/gender; race/ethnicity; education level; occupation; language; income	Perception; understanding
35	Davis et al. 1998 [61]	USA	Quantitative	Adults recruited from private and university oncology clinics and a low-income housing complex	Age; sex/gender; race/ethnicity; education level	Comprehension; attitude
36	de Oliveira et al. 2017 [62]	USA	Quantitative	Participants were recruited from Amazon’s Mechanical Turk (mturk)	Age; sex/gender; race/ethnicity	Comprehension
37	Dellson et al. 2019 [63]	Sweden	Quantitative	Patients who could read and speak Swedish and who were eligible for 23 selected clinical cancer trials were included. [Physicians recruiting them to an RCT]	Age; sex/gender; education level; previous clinical trial participation	Knowledge; understanding
38	Diemert et al. 2017 [64]	Brazil and USA	Quantitative	Healthy adults participating in clinical trials of the Na-GST-1/Alhydrogel hookworm vaccine	Age; sex/gender; education level; employment status; health insurance status; previous clinical trial participation	Knowledge; attitude
39	Ditai et al. 2018 [65]	Uganda	Mixed methods	Women of at least 34 weeks’ pregnancy, suitable for inclusion in the babygel pilot trial	Age; sex/gender; language of administration; marital status; education level; occupation	Recollection; understanding
40	Dresden et al. 2001 [66]	USA	Quantitative	100 patients with a history of asthma were seen in an urban, county teaching emergency department (ED)	Age; sex/gender; race/ethnicity; language; educational level; employment status	Recollection
41	Duvall Antonacopoulos et al. 2016 [67]	Canada	Quantitative	18 years of age or older who were recruited through Mechanical Turk (mturk), a website that is managed by the company Amazon	Age; sex/gender; education level; income	Comprehension
42	Eichner et al. 2020 [68]	Germany	Mixed methods	Hospitalised first-ever ischaemic stroke patients who took part in the informed consent procedure of the SANO trial	Age; sex/gender; education level	Understanding
43	Falvo et al. 2021 [69]	Switzerland	Qualitative	Participants of the validation phase of an epidemiological study on the prevalence and impact of dementia in Switzerland. Participants were older adults (65 years or more) eligible for the dementia study and their informant	Age; sex/gender; nationality; location; education level; occupation	Comprehension
44	Ford et al. 2008 [70]	USA	Quantitative	Parkinson disease patients and their caregivers who had an appointment at the Parkinson Disease clinic	Age; sex/gender; race/ethnicity; education level; income; marital status	Comprehension; recollection
45	Fortney et al. 1999 [71]	Countries in Latin America, Africa, and USA	Qualitative	Women who participated in clinical trial undertaken to assess the effectiveness of a barrier contraceptive	Age; sex/gender; marital status; education level; location	Perception; recollection
46	Freeman et al. 2013 [72]	USA	Quantitative	Prospective living liver donors (lds) eligible for A2ALL study	Age; marital status; education level; location	Understanding
47	Gad et al. 2022 [73]	Denmark	Mixed methods	Patients at their first visit to the phase 1 unit for cancer trial	Age; sex/gender; cohabitation; education level; employment status	Understanding
48	Gammelgaard et al. 2004 [74]	Denmark	Mixed methods	181 patients (103 who gave informed consent and 78 who did not consent to the second Danish acute myocardial infarction trial (DANAMI-2))	Age; sex/gender	Understanding
49	Gertsmen et al. 2020 [75]	Canada	Mixed methods	English- and French-speaking legal guardians of children less than 18 years old, who had been admitted to the PICU within the past 24 h and were expected to stay at least 48 h	Age; education level; previous clinical trial participation	Understanding
50	Ghormley et al. 2011 [76]	USA	Quantitative	Inpatients diagnosed with major depression were recruited from a hospital psychiatric unit, and normal controls	Age; sex/gender; race/ethnicity; education level; language	Understanding
51	Gillespie 2017 [77]	USA	Quantitative	Adult cardiology clinical trial participants	Age; gender; education level; income; previous clinical trial participation	Understanding
52	Goldberger et al. 2011 [78]	USA	Quantitative	Patients undergoing an initial diagnostic cardiac electrophysiology study	Age; sex/gender; education level	Comprehension; satisfaction
53	Golembiewsky et al. 2021 [79]	USA	Quantitative	Adult patients were recruited from 4 family medicine clinics in Florida	Age; sex/gender; race/ethnicity; education level	Knowledge; understanding
54	Gota et al. 2018 [80]	India	Quantitative	Cancer patients enrolled in phase 1, 2, or 3 interventional clinical trials	Age; sex/gender; education level	Quality of IC process
55	Griffin et al. 2006 [81]	USA	Quantitative	Male veterans with heart disease and low levels of high-density lipoprotein (HDL) cholesterol who participated in a clinical trial	Age; marital status; race/ethnicity; education level	Knowledge
56	Guarino et al. 2006 [82]	USA	Quantitative	Participants of a ‘parent’ 2 × 2 factorial randomised clinical trial of exercise and cognitive behavioural therapy (CBT) for the treatment of Gulf War veterans’ illnesses (CSP #470)	Age; sex/gender; race/ethnicity; education level; employment status	Understanding; satisfaction
57	Guarino et al. 2006 [83]	USA	Quantitative	Participants of a ‘parent’ 2 × 2 factorial randomised clinical trial of exercise and cognitive behavioural therapy (CBT) for the treatment of Gulf War veterans’ illnesses (CSP #470)	Age; sex/gender; race/ethnicity; education level	Understanding; satisfaction
58	Harrison et al. 1995 [84]	USA	Quantitative	Participants recruited in HIV vaccination subunit trial	Sex/gender	Comprehension
59	Hlubocky et al. 2018 [85]	USA	Mixed methods	Advanced cancer patients who participate in phase I clinical trials	Age; sex/gender; race/ethnicity; education level; income	Communication
60	Hoffner et al. 2012 [86]	USA	Quantitative	Adult cancer patients considering participation in a clinical trial	Age; sex/gender; race/ethnicity; education level; marital status; previous clinical trial participation	Understanding
61	Hofmeijer et al. 2007 [87]	The Netherlands	Quantitative	Legal representatives of patients who participated in two clinical trials (HAMLET and PAIS)	Age	Recollection
62	Howard et al. 1981 [88]	USA	Mixed methods	Participants in the Beta-blocker Heart Attack Trial (BHAT)	Age; sex/gender; race/ethnicity; education level; marital status	Understanding
63	Hu et al. 2022 [89]	China	Quantitative	Participants of clinical trials who consented to be contacted for clinical trial recruitment	Age; sex/gender; race/ethnicity; occupation; education level; income	Knowledge; attitude
64	Hughes et al. 2017 [90]	UK	Quantitative	Adults with a history of back pain	Age; sex/gender; race/ethnicity; occupation; education level; previous clinical trial participation	Knowledge
65	Hutchison et al. 2007 [91]	UK	Mixed methods	Patients with cancer who had no experience of clinical trials, patients with cancer who had previously participated in an RCT, and research nurses	Age; sex/gender; education level; socio-economic status; clinical trial participation	Knowledge; understanding
66	Jefford et al. 2011 [92]	Australia	Quantitative	Patients were eligible for the consent study if they gave signed informed consent and had enrolled on a suitable therapeutic CCT within the preceding 2 weeks	Age; sex/gender; marital status; employment status; country of birth; language; education level	Understanding
67	Jeong et al. 2012 [93]	Korea	Quantitative	Patients and nurses who could participate in biomedical research as subjects or research assistants	Age; sex/gender; education level; occupation; previous participation in biomedical research	Understanding
68	Joffe et al. 2001 [94]	USA	Quantitative	Adult patients with cancer who had recently enrolled in a clinical trial at one of three affiliated institutions	Age; sex/gender; race/ethnicity; marital status; language; education level	Knowledge
69	Joffe et al. 2001 [18]	USA	Quantitative	Subjects were eligible if they were greater than or equal to 18 years old and had signed an informed consent form to a qualified cancer clinical trial	Age; sex/gender; race/ethnicity; educational level	Understanding
70	Juan-Salvadores et al. 2022 [95]	Spain	Quantitative	Patients enrolled in ongoing clinical trials at four medical departments (Hemodynamics, Electrophysiology, Oncology, Internal Medicine) in two hospitals in Spain	Age; sex/gender; marital status; language; education level; employment status	Understanding
71	Juraskova et al. 2008 [96]	Australia	Mixed methods	Post-menopausal women participating in follow-up for the IBIS-I breast cancer prevention trial	Age; marital status; education level; occupation; nationality; previous clinical trial participation	Knowledge; understanding; satisfaction; attitude
72	Karlawish et al. 2002 [97]	USA	Qualitative	Patients with mild to moderate Alzheimer’s disease, their family caregivers, and age/education-matched non-demented older persons	Age; education level	Understanding
73	Kashur et al. 2023 [98]	Canada	Mixed methods	Patients enrolled in 3 ongoing acute myocardial infarction (AMI) randomised controlled trials	Age; sex/gender; language; education level; previous clinical trial participation	Understanding
74	Kass et al. 2009 [99]	USA	Quantitative	Cancer patients with possible participation in an early-phase clinical trial	Age; education level; income; race/ethnicity; previous clinical trial participation	Understanding
75	Kass et al. 2015 [100]	USA	Quantitative	Participants enrolling in affiliated ongoing clinical trials	Age; sex/gender; race/ethnicity; income; employment status; health literacy; medical insurance status; previous clinical trial participation	Understanding
76	Kim & Kim 2015 [101]	South Korea	Quantitative	Main family caregivers of cancer patients who were 18 years or older, not already participating in clinical trials, and able to read and understand Korean	Age; sex/gender; education level; income; previous clinical trial participation; health literacy	Understanding
77	Knapp et al. 2009 [102]	UK	Quantitative	Men aged 18–40 years, to match the volunteers in the actual TGN1412 trial	Age; sex/gender; educational level; occupation	Understanding
78	Knapp et al. 2009 [103]	UK	Mixed methods	Participants were adult women aged under 45, as in the actual ‘Poor Responders’ IVF trial	Age; sex/gender; education level; employment status	Understanding
79	Koh et al. 2012 [104]	South Korea	Quantitative	Participants in clinical trials performed at Seoul National University Hospital	Age; sex/gender; education level; previous clinical trial participation	Understanding
80	Koonrungsesomboon et al. 2017 [105]	Thailand	Quantitative	Participants from 8 clinical trials at Thammasat University Hospital	Age; sex/gender; education level	Understanding
81	Kripalani et al. 2008 [106]	USA	Quantitative	Patients with coronary heart disease enrolling in a randomised controlled trial at an inner-city teaching hospital in Atlanta, GA	Age; sex/gender; race/ethnicity; marital status; employment status; education level	Comprehension
82	Krosin et al. 2006 [107]	Mali	Quantitative	Participants who had given informed consent for their children to participate in a malaria vaccine trial in two villages in Mali	Sex/gender; education level; literacy status; location; income	Comprehension
83	Kruse et al. 2000 [108]	Denmark	Quantitative	Adult outpatients (≥ 18 years) attending Medical Gastroenterology, Gynecology, Orthopedic Surgery, and Urology clinics at H:S Hvidovre University Hospital	Age; sex/gender; language; educational level	
84	Länsimies-Antikainen et al. 2010 [109]	Finland	Quantitative	Subjects aged 57–78 years, who are participating in a 4-year randomised controlled intervention trial on the effects of physical exercise and diet on atherosclerosis, endothelial function and cognition	Age; sex/gender; marital status; education level; employment status; occupation; previous clinical trial participation	Understanding
85	Leach et al. 1999 [110]	The Gambia	Qualitative	The Gambian population who were asked to give consent for a vaccine trial	Age; sex/gender; location; race/ethnicity; education level	Attitude
86	Lewis 2015 [111]	USA	Mixed methods	Participants in HIV trials	Age; sex/gender; race/ethnicity; education level; health literacy; socio-economic status	Understanding
87	MacQueen et al. 2014 [112]	Tanzania	Quantitative	Women eligible for HIV-prep Clinical Trial Study	Age; sex/gender; education level; religion; marital status	Comprehension
88	Mansour et al. 2015 [113]	Egypt	Qualitative	103 individuals participating in 10 different therapeutic clinical trials at the Clinical Research Centre	Age; sex/gender; marital status; employment status; education level	Perception
89	Mboizi et al. 2017 [114]	The Gambia	Quantitative	Parents of infants participating in a pneumococcal vaccine trial in a peri-urban setting in The Gambia	Age; sex/gender; occupation; education level; religion	Knowledge; recollection
90	Meneguin et al. 2014 [115]	Brazil	Qualitative	Patients who participated in clinical trials about hypertension and coronary disease in a specialised cardiologic hospital located in the city of Sao Paulo	Age; sex/gender; education level; literacy level	Perception; understanding
91	Mexas et al. 2014 [116]	Brazil	Quantitative	Patients with pulmonary TB who enrolled in the riomar study (‘Randomised, Open-Label Trial of a Rifapentine Plus Moxifloxacin-Based Regimen for Intensive Phase Treatment of Smear-Positive Pulmonary Tuberculosis’)	Age; sex/gender; education level; income; location	Recollection; understanding
92	Miller et al. 1994 [117]	USA	Quantitative	Patients from a trial on OTC analgesic use	Age; sex/gender; race/ethnicity; education level	Understanding
93	Miller et al. 1996 [118]	USA	Quantitative	Adults entering one of four prospective, randomised, double-blind, multicentre, ambulatory trials of antiinfective agents	Age; sex/gender; education level; literacy level	Perception; recollection
94	Miller et al. 2013* [119]	USA	Mixed methods	Parents who were considering enrolling a child in a phase I study at six children’s hospitals in the USA	Age; sex/gender; race/ethnicity; socio-economic status	Comprehension
95	Mills et al. 2003 [120]	UK	Qualitative	Participants eligible for trial of treatments for localised prostate cancer (Prostate testing for cancer and Treatment—protect study)	Age; sex/gender	Perception
96	Minnies et al. 2008 [121]	South Africa	Quantitative	Mothers who gave informed consent for the participation of their children in the immunology study nested within a tuberculosis vaccine trial	Age; language; education level	Recollection; understanding
97	Montgomery et al. 1998 [122]	UK	Quantitative	Parents of patients who had participated in one of six clinical trials in anaesthesia	Age; sex/gender	Recollection; satisfaction
98	Moodley et al. 2005 [123]	South Africa	Quantitative	Participants from two influenza vaccine trials conducted in impoverished non-white communities outside Cape Town	Age; sex/gender; race/ethnicity; education level; language	Recollection
99	Nguyen et al. 2023 [124]	Vietnam	Qualitative	Study physicians and trial participants from two clinical trials conducted at the Oxford University Clinical Research Unit and Hospital for Tropical Diseases in Ho Chi Minh City	Age; sex/gender; education level; location	Perception; understanding
100	Norris et al. 1990 [125]	USA	Quantitative	Potential participants who volunteered to screen consent form and fill the 14-item, MCQ exam before signing consent form	Not reported	Knowledge; understanding
101	O’Sullivan et al. 2022 [126]	Ireland	Quantitative	Adults ≥ 18 years who are HIV-positive, on stable HIV treatment with a suppressed viral load, and have obesity or overweight with comorbidities	Not reported	Understanding
102	Ormond et al. 2009 [127]	USA	Qualitative	Participants in the nugene biobank project at Northwestern University	Age; sex/gender; race/ethnicity; education level; income; employment status; religion; relationship status	Comprehension; recollection
103	Ossemane et al. 2018 [128]	Mozambique	Quantitative	The parents/guardians who were approached to enrol their child in the primary bacteraemia study	Age; sex/gender; language; education level	Quality of IC process
104	Paris et al. 2015 [129]	France	Quantitative	Adult patients undergoing screening for enrolment in biomedical research studies	Age; sex/gender; educational level; occupation; previous clinical trial participation; socio-economic class	Comprehension
105	Penn et al. 2010 [130]	South Africa	Mixed methods	19 students from two English-speaking universities who were recruited as volunteers	Age; sex/gender; language; education level; occupation	Understanding
106	Ponzio et al. 2018 [131]	Italy	Quantitative	Subjects with multiple sclerosis were recruited from the Rehabilitation Center of the Italian MS Society and had not previously participated in either of the two studies being evaluated	Age; sex/gender; education level	Understanding
107	Pope et al. 2003 [132]	Canada	Quantitative	Participants from 14 clinical trials in rheumatology, ophthalmology, and cardiology at the University of Western Ontario	Age; sex/gender; race/ethnicity; education level; previous clinical trial participation	Satisfaction
108	Ranjan et al. 2019 [133]	India	Quantitative	Volunteers participating in clinical trials in contract research organisations in Delhi, India	Age group; sex/gender; race/ethnicity; marital status; educational level; income; occupation; location; religion; previous clinical trials participated	Knowledge; attitude
109	Ravina et al. 2010 [134]	USA and Canada	Quantitative	Subjects enrolled in an NIH sponsored, phase II Parkinson’s disease clinical trial	Age; sex/gender; race/ethnicity; education level	Comprehension; satisfaction
110	Rikkert et al. 1997 [135]	The Netherlands	Quantitative	Frail elderly patients admitted to the Department of Geriatric Medicine	Age; sex/gender; educational level	Comprehension
111	Rose et al. 2013 [136]	UK, Denmark, Germany, and Poland	Qualitative	Participants who had taken part in the GENDEP pharmacogenetic trial of antidepressant medication	Age; sex/gender; race/ethnicity; education level	Understanding
112	Roth et al. 2021 [137]	USA	Quantitative	Patients with previously treated advanced non-small cell lung cancer enrolled in the Lung Cancer Master Protocol (Lung-MAP) trial	Age; sex/gender; race/ethnicity; education level; income	Knowledge; understanding
113	Ruiz de Hoyos et al. 2020 [138]	Spain	Quantitative	Adult patients participating in one of the drug-based clinical trials our hospital is involved in and for which they had been asked to fill in an IC form in the previous 30 days	Age; sex/gender; education level; employment status; language; previous clinical trial participation	Perception; understanding
114	Russel et al. 2005 [139]	Australia	Mixed methods	20 Aboriginal and 20 non-Aboriginal women in Alice Springs	Age; sex/gender; race/ethnicity; employment status; education level; language	Understanding; recollection
115	Sanchini et al. 2014 [140]	Italy	Quantitative	77 cancer patients previously enrolled in randomised phase II or phase III clinical trials	Age; sex/gender; education level	Comprehension
116	Sand et al. 2008 [141]	Norway	Qualitative	Patients eligible for a randomised phase III trial of chemotherapy for advanced non-small cell lung cancer	Age; sex/gender; education level	Understanding
117	Sarkar et al. 2010 [142]	India	Quantitative	Parents of malnourished children participating in a nutritional supplementation trial	Age; sex/gender; religion; education level; socio-economic status; occupation	Comprehension
118	Schats et al. 2003 [143]	The Netherlands	Quantitative	Patients with subarachnoid haemorrhage who had participated in one of two randomised clinical trials, and their relatives	Trial participation status	Recollection; understanding
119	Schmanski et al. 2021 [144]	USA	Quantitative	Participants enrolled in the Colorado Center for Personalized Medicine (CCPM) Biobank	Age; sex/gender; race/ethnicity; income; education level; prior participation; employment	Knowledge; recollection
120	Schumacher et al. 2017 [145]	USA	Quantitative	Patients were eligible if they were 18 years of age or older, English-speaking (though not necessarily as the first language), and if they had agreed to participate in a trial of cancer therapy at the Rhode Island Hospital Comprehensive Cancer Centers (RIHCCC)	Age; sex/gender; race/ethnicity; education level; language	Understanding
121	Searight et al. 1996 [146]	USA	Qualitative	Adults who had completed one of two clinical drug studies (herpes labialis or genital herpes trials)	Age; sex/gender; race/ethnicity; education level	Perception; understanding
122	Sengupta et al. 2011 [147]	USA	Quantitative	Newly enrolled HIV-infected subjects in Adult AIDS Clinical Trial Group (AACTG) protocols	Age; sex/gender; race/ethnicity; marital status; education level; language	Understanding
123	Shafiq et al. 2011 [148]	India	Quantitative	Cardiology outpatients invited to participate in a multicentric phase III, randomised controlled trial	Education level; socio-economic status	Comprehension
124	Shamy et al. 2019 [149]	Canada and Europe	Quantitative	Patients enrolled in the ESCAPE trial using deferred consent, or their authorised third parties	Age; sex/gender; race/ethnicity; country of origin	Knowledge
125	Shelton et al. 2015 [150]	USA	Quantitative	Adult visitors to ICU waiting rooms	Age; sex/gender; race/ethnicity; education level; previous research participation	Understanding
126	Shiono et al. 2014 [151]	Japan	Quantitative	Healthy Japanese women aged 40–49 participating in the J-START breast cancer screening trial	Age; educational level; marital status; employment status	Understanding
127	Siao et al. 2014 [152]	USA	Quantitative	Outpatients undergoing endoscopy at San Francisco General Hospital and Trauma Center	Age; race/ethnicity; education level; previous endoscopic procedure participation; language	Recollection; satisfaction
128	Smith and Fogarty 2016 [153]	Australia and New Zealand	Quantitative	Women eligible to participate in the clinical trial are aged less than 43 years undergoing a fresh IVF or intracytoplasmic sperm injection (ICSI) cycle, and not currently receiving acupuncture	Age; location; education level	Communication; understanding
129	Spellecy et al. 2011 [154]	USA	Mixed methods	Adult patients potentially eligible for BMT CTN clinical trials	Age; sex/gender; education level	Comprehension; satisfaction
130	Sudore et al. 2006 [155]	USA	Qualitative	Two hundred and four ethnically diverse subjects aged ≥ 50, consenting for a trial to improve the forms used for advance directives	Age; sex/gender; race/ethnicity; income; education level; literacy level; language; country of birth	Comprehension
131	Tadros et al. 2019 [156]	Australia	Quantitative	Healthy volunteers participating in phase I clinical trials	Age; sex/gender; race/ethnicity	Comprehension; recollection
132	Tait et al. 2003 [157]	USA	Mixed methods	Parents or guardians who had been approached to allow their child to participate in 1 of 18 ongoing clinical anaesthesia or surgery studies	Parent’s age; child’s age; sex of child; race/ethnicity; education level; income; previous research participation (parent/child)	Understanding
133	Taiwo and Kass 2009 [158]	Nigeria	Qualitative	Adults were eligible who had just provided consent (within the last hour) to one of three ongoing independent oral health research projects (called here ‘parent studies’) and who were willing to undergo a post-assessment interview	Age; sex/gender; marital status; educational level; location; previous research participation	Knowledge; understanding
134	Taylor et al. 2021 [159]	USA	Quantitative	Participants eligible to enrol in six actual clinical trials (referred to as ‘parent studies’)	Age; sex/gender; race/ethnicity; education level; income; employment status; marital status	Understanding; satisfaction
135	Tindall et al. 1994 [160]	Australia	Quantitative	113 subjects with AIDS or AIDS-related complex who were intolerant of zidovudine and enrolling in a clinical trial of didanosine (ddi)	Age; sex/gender; sexual orientation; education level; previous clinical trial participation	Knowledge
136	van den Bergh et al. 2009 [161]	The Netherlands and Belgium	Quantitative	High-risk subjects eligible for lung cancer CT screening trial (NELSON trial)	Age; sex/gender; educational level; employment status	Knowledge; perception; attitude
137	van Stuijvenberg et al. 1998** [162]	The Netherlands	Quantitative	Parents who volunteered their child (230 children) for a randomised, double-blind, placebo-controlled trial of ibuprofen syrup to prevent recurrent febrile seizures	Age (parents); sex/gender (parents); race/ethnicity; marital status; education level; occupation; language	Communication; perception; understanding
138	Verheggen et al. 1996 [163]	The Netherlands	Quantitative	Adult patients approached for clinical trials at the University Hospital in Maastricht	Age; sex/gender; educational level; marital status; religion; employment status; previous clinical trial participation	Perception
139	Vickers et al. 2021 [164]	USA	Quantitative	Patients undergoing prostate biopsy as part of active surveillance for low-risk prostate cancer	Age; sex/gender	Knowledge; understanding
140	Wade et al. 2009 [165]	UK	Qualitative	Purposive sample of 23 recruitment appointments from three study centres	Age; sex/gender; race/ethnicity; marital status; education level; employment status; previous research participation	Quality of IC process; communication; understanding
141	Wada et al. 2017 [166]	UK	Mixed methods	Patients participating in recruitment appointments for randomised controlled trials	Not reported	Communication; understanding
142	Weston et al. 1997 [167]	Canada	Quantitative	English-speaking women between 20 and 32 weeks of gestation (ineligible for term PROM study)	Age; education level; language	Knowledge
143	Wirshing et al. 1998 [168]	USA	Quantitative	49 schizophrenic patients participating in ongoing clinical treatment research trials	Age; sex/gender; marital status; race/ethnicity; education level	Comprehension
144	Woodward et al. 1979 [169]	USA	Quantitative	Volunteers are solicited from the Baltimore metropolitan area by means of newspaper advertisements, posters, and discussions with previous participants	Age; education level; previous research participation	Examination
145	Yanics et al. 1996 [170]	USA	Quantitative	The subjects were enrolled in their respective clinical drug trial designed to assess the efficacy of a new drug in the treatment of either genital herpes, herpes labialis (cold sores), or vaginal yeast infection	Age; sex/gender; marital status; education level; employment status; language	Comprehension; recollection
146	Young-Afat et al. 2021 [171]	The Netherlands	Quantitative	Participants in the colorectal cancer, bone metastases, and breast cancer cohorts at the Department of Radiation Oncology of the University Medical Center Utrecht	Age	Understanding; recollection
147	Yuval et al. 2000 [172]	Israel	Quantitative	Patients thought to be within 24 h of the onset of symptoms of suspected acute myocardial infarction	Age; language	Perception; comprehension
148	Zhang et al. 2017 [173]	China	Quantitative	Age-related cataract patients scheduled for their first elective cataract surgeries between July 2014 and March 2015 were recruited for this study. All the patients invited to participate in the study were deemed capable of informed consent and capable of watching a video (i.e. they had a best-corrected visual acuity [BCVA] of more than 1.0 [logmar chart] for either eye)	Age; sex/gender; language; education level	Understanding

^*^Included patients aged 14–21 years, therefore included in this review

^**^Measure completed by parents of participating children, therefore included as completed by adult

#### Country and study design

Among the 148 studies, 37.2% originated in the USA (55/148), 6.8% in the UK (10/148), and 56.0% in other countries. Of all studies, 70.3% (104/148) were quantitative, 16.2% (24/148) mixed-methods, and 13.5% (20/148) qualitative (see Table 1).

#### Age

Age was reported for 88% (130/148) of the studies (details in Supplementary Table 2).

Included studies were conducted among adult participants of a wide range of ages across studies, from young adults (e.g. 17.8 years) [119] to older adults (e.g. 80.1 years) [135]. This study [119] was retained because the assessment approach was not specifically designed for paediatric populations and comprised a mixed adolescent and adult sample (ages 14–21 years), with the majority of participants aged 18–21 years and consented as adults. The mean age distribution was clustered around middle age (median of reported mean ages: 46.6 years), calculated from the 110 studies that reported a mean age; the remaining studies reported age using medians, ranges, or categorical groupings. Twenty-eight studies (19%) reported comparisons across participant groups, for example, intervention vs control [31], younger vs older group [50], or stakeholder categories such as patients vs physicians [63].

#### Level of education

Education was among the most reported PRO EDI characteristics (124/148; 83.8%). The categories used for reporting education level differed across the studies, reflecting diversity in a research context and made it difficult to make comparisons between studies. The level of education ranged from basic literacy to postgraduate education (see Supplementary Table 2). Despite its frequency, education was typically used descriptively rather than analytically.

#### Language

Out of the 148 included studies, only 28 (18.9%) reported the language used by participants or the language in which questionnaires and consent materials were administered. In these studies, language was mentioned as a simple descriptor, for example, when listing socio-economic demographic factors, or when indicating whether consent was obtained in the participant’s language, but outcomes were not analysed by language. The infrequent reporting suggests a potential under-recognition of the impact of linguistic barriers in clinical trial participation.

#### Race/ethnicity

Race/ethnicity was the most frequently reported among the included studies (60/148; 40.5%) after age, sex/gender, and education. Yet the fact that fewer than half of the studies reported it highlights the ongoing gaps in recognising the need to account for ethnic diversity when evaluating IC.

### Constructs and timing of assessment

#### Constructs assessed

As displayed in Table 1, although all studies aimed to assess “understanding,” they deployed a heterogeneous set of constructs (e.g. comprehension, knowledge, recall, perception, satisfaction). The wide array of terms adopted by different authors underscores a lack of consensus around what constructs should be used to measure participants’ understanding.

#### Domains assessed

There were many domains assessed to evaluate the target constructs identified by each study, and these are shown in Supplementary Table 2.

#### Initial measure

In around one-third of the studies, participants’ understanding was evaluated after they consented to the clinical trial. Some studies did not specify the timepoint of evaluation [11, 28, 33, 35, 36, 40, 44, 46–48, 53, 61, 77, 81, 89, 91, 96, 104, 109, 114, 115, 133, 136, 144–146, 153], while others reported ‘immediate’ assessment [30, 31, 73, 78, 79, 113, 118, 119, 121, 129, 130, 151, 159, 170, 173]. Others measured within a defined timeframe after consent, such as within 24–72 h [65, 68, 75, 107, 116, 142, 164], within 1–2 weeks [18, 29, 42, 45, 70, 80, 86, 92, 120, 154], within 1–2 months [43, 54, 71, 117, 124], or after more extended periods [41, 56, 87, 88, 122, 123, 132, 134, 140, 143]. Assessing retention of information after an extended period may provide insight into long-term understanding, though other factors beyond the initial consent process may influence results. This variability reflected differing views of the authors on the optimal timing for assessing understanding.

Interestingly, very few studies assessed understanding during recruitment [50, 52, 55, 63, 84, 97, 100, 128, 139, 166], or after specific recruitment steps, such as after reading the information sheet [49, 66, 90, 93, 101–103, 155], or following educational intervention [72, 95, 150]. These approaches isolated the impact of particular recruitment components. Twelve studies assessed understanding before consent was given [37, 85, 101, 106, 125, 131, 135, 148, 156, 167–169], which allowed them to correct misunderstandings before formal enrolment.

In some cases, although participants were enrolled, the intervention was delayed until understanding was measured [39, 86, 92, 110, 126, 152, 158, 160, 161, 164]. Some of these trials involved invasive procedures such as chemotherapy or surgery, making comprehension critical before intervention. One study [171] assessed understanding at multiple points, providing a more comprehensive view of how comprehension evolved during participation. However, two studies [32, 165] did not report the stage at which understanding was assessed.

The variability in assessment timing suggests a lack of standardisation in how understanding for IC is currently evaluated.

#### Subsequent (recall) measures

In 37 studies, understanding was re-evaluated at different times afterwards, sometimes referred to as recall. Some evaluated recall immediately after giving the trial information [160], or within 24 h [128]; others evaluated retention at 1 year [83, 129]. Most studies used a recall period of 1–4 weeks [30, 31, 55, 57, 70, 79, 126, 135, 139, 167, 168], or 2–6 months [34, 49, 52, 54, 58, 79, 83, 114, 116, 147, 149, 169], whereas other studies had follow-up over a varied range of periods [42, 53, 71, 170]. Six studies mentioned a follow-up to assess recall of information, but the exact timing was not specified [39, 50, 51, 85, 98, 162].

### Tool/measure characteristics

Supplementary Table 3 presents the characteristics of the tools/measures used in the study to measure their constructs and domain. Across the included studies, a wide range of tools were used to assess informed consent, which can be broadly grouped into four categories. First, standardised questionnaires were commonly used, most notably the Quality of Informed Consent (QuIC) [18, 29, 40, 44, 57, 73, 77, 79, 80, 86, 92, 94, 96, 129, 154, 164] and its modified versions [33, 38, 45, 48, 65, 70, 101, 104, 127, 138, 145, 147, 158], assessing objective and subjective understanding of core consent elements (e.g. study purpose, risks, benefits, voluntariness). Second, study-specific or ad hoc comprehension quizzes were frequently employed to test recall and understanding of trial concepts such as randomisation, placebo, and alternatives to participation [36, 52, 59, 66, 140]. Third, structured or semi-structured interviews were used to explore participants’ understanding, decision-making processes, and perceptions of the consent experience in greater depth [41, 54, 136, 141–143, 146]. Finally, a smaller number of studies used observational or interaction-based approaches, including audio- or video-recorded consent encounters and interactional analyses, to assess how consent information was conveyed and understood in practice [42, 85]. Overall, tool selection varied by study context, population, and research design.

#### PPIE involvement

Among the 148 studies, only 20 reported involving diverse stakeholders such as physicians, patients, or a mixed panel. Table 2 demonstrates that although PPIE was incorporated at different stages, its primary focus was instrument development and refinement. The level of PPIE involvement also varied, with some extensive collaboration in the designing stages [54, 72, 91, 100], whereas others utilised PPIE for getting feedback on specifically targeted components [46, 83, 90, 104, 109, 134, 138]. None of the studies reporting on PPIE discussed the characteristics of PPIE contributors, which limits our understanding of how inclusive the groups were. This makes it difficult to determine whether diverse perspectives were accounted for when developing these tools or measures.

**Table 2:** PPIE involvement in tool/measure development

Tool and study	Study type	PPIE activity (verbatim)	Type of PPIE contributors involved
Beranger et al. 2019 [43]	Tool validation	Stakeholder input used to elaborate and validate interview and questionnaire frameworks	Psychologists, physicians, and parents representing the patient association
Bhansali et al. 2009 [46]	Tool development	Administration of draft questionnaire to laypersons and incorporation of feedback before finalisation	Six laypersons
Chappuy et al 2010 [54]	Tool development	Collaboration in the design of the semi-structured interview framework	Psychologists, members of the parents’ association, and investigating physicians
da Fonseca et al. 1999 [59]	Tool development	Pilot testing of the questionnaire and incorporation of feedback	Members of a self-help NGO for HIV-positive people
Freeman et al. 2013 [72]	Tool development	Collaborative development of scripted educational content and assessment modules	Members of A2ALL consortium, expert in competence assessment (Paul Appelbaum, MD)
Gertsman et al. 2020 [75]	Tool development	Identification of key consent concepts and construction of questionnaire items through stakeholder consultation	Parents of PICU patients
Guarino et al. 2006 [83]	Tool development and validation	Review and modification of the draft questionnaire by a consumer focus group	A focus group of five Gulf War veterans
Hughes et al. 2017 [90]	Tool development	Pretesting of a newly constructed placebo knowledge questionnaire and modification based on feedback	10 lay volunteers
Hutchison et al. 2007 [91]	Tool development and validation	Direct patient involvement in questionnaire design, review of drafts, and feedback during pilot testing	Four patients
Juraskova et al. 2008 [96]	Tool development	Consumer review of decision aid content and structured feedback via questionnaires and semi-structured interviews	ANZ BCTG Consumer Advisory Panel
Kass et al. 2015 [100]	Tool application	Focus groups and cognitive interviews informed development of a consent understanding assessment tool (CUE); interventions then applied in real trials	Current and former research participants, community advisory board members
Koh et al. 2012 [104]	Tool application	Pilot testing of a culturally adapted questionnaire to ensure readability and acceptability	10 people not related to the study
Koonrungsesomboon et al. 2017 [105]	Tool application	Independent laypersons reviewed SIDCER ICFs and post-test questionnaires to improve readability and understandability	Collaborating investigators and independent laypersons
Krosin et al. 2006 [107]	Tool development	Questionnaire content developed through consultation with local IRB members, village leaders, and investigators	Malian IRB
Länsimies-Antikainen et al. 2010 [109]	Tool application	Questionnaire previously tested in a pilot study; administered to participants to assess comprehension	Five previous study participants
Ravina et al. 2010 [134]	Tool validation	Pilot testing and face validity assessment of a newly developed comprehension questionnaire with individuals with Parkinson’s disease not enrolled in the trial	Five individuals with PD not enrolled in the study
Roth et al. 2021 [137]	Tool development	Cognitive debriefing and pilot testing used to refine survey items and response formats prior to full deployment	SWOG Lung Committee patient advocate
Ruiz de Hoyos et al. 2020 [138]	Tool development and validation	Pilot testing of the draft questionnaire with patients; collection of feedback on clarity, usability, and acceptability leading to item modification and optimisation	Patients (through personal interviews)
Russel et al. 2005 [139]	Tool application	Aboriginal participants evaluated consent materials through questionnaires, interviews, and group discussions; feedback used to identify weaknesses and preferences	Aboriginal staff members
Sengupta et al. 2011 [147]	Tool application	Community Advisory Board reviewed and pretested the adapted QuIC questionnaire; participants’ responses used to evaluate effectiveness of the intervention	Local AACTG’s Community Advisory Board

### Comparison across tools/measures

There were 16 tools/measures that had some aspect of both reliability and validity reported. Although the DICCQ, PIC, and P-QIC demonstrated the strongest psychometric properties according to COSMIN criteria (Table 3), their validation contexts were limited. The DICCQ was primarily validated in a low-literacy population within a single country, whereas the P-QIC was developed and evaluated using simulated consent scenarios with volunteer actors rather than real trial participants.

**Table 3:** Psychometric properties evaluation based on COSMIN criteria

Tool name	Internal consistency	Test–retest reliability	Inter-rater reliability	Content validity	Construct validity	Overall quality of evidence
QuIC Questionnaire [18]	(?)	(±) ICC: 0.66–0.77	(?)	(+) Expert consensus on federal regulations	(?)	Low—limited evidence, small number of properties tested
Deaconess Informed Consent Comprehension Test (DICCT) [118]	(?)	(?)	(+) *r* = 0.84	(?)	(+) correlations with WAIS-R and WRAT-R	Moderate—good reliability and construct validity evidence
DICCQ [30]	(+) *α* = 0.73–0.79	(+) ICC = 0.94	(?)	(+) Face and content validity with piloting	(+) multiple validity types demonstrated	High—comprehensive validity testing and good reliability
Informed Consent Questionnaire (ICQ-4) [83]	(-) *α* < 0.70	(?)	(?)	(?)	(+) factor analysis and discriminant validity	Low—poor internal consistency
Questionnaire developed for the study [163]	(±) *α* = 0.60–0.90	(?)	(?)	(?)	(+) factor analysis	Low—limited evidence
The Electronic Informed Consent (eIC) Attitudes Scale [89]	(+) *α* = 0.806	(?)	(?)	(+) Expert checked	(?)	Low—limited evidence
Patient Understanding of Research [91]	(+) *α* = 0.78	(?)	(?)	(+) Expert input and reassessment	(?)	Low—limited evidence
13-item post-test instrument [150]	(+) *α* = 0.73	(?)	(?)	(+) Content analysis table and experts	(?)	Low—limited evidence
PIC measure [166]	(?)	(+) > 90% agreement	(+) > 90% agreement	(+) Based on guidelines and expert review	(+) multiple validity types	High—comprehensive testing
MacArthur Competency Assessment Tool for Clinical Research (MacCAT-CR) [97]	(?)	(?)	(+) ICC = 0.75–0.99	(?)	(+) multiple validity evidence	Moderate—strong validity but limited reliability data
MacLiver [72]	(?)	(?)	(+) 90% agreement	(+) Based on established MacCAT-CR	(?)	Low—limited evidence
The Understanding Treatment Disclosures (UTD) Scale [76]	(?)	(?)	(+) ICC = 0.87–0.96	(?)	(+) convergent and criterion	Moderate—strong where tested
P-QIC [55]	(+) *α* = 0.98	(±) *r* = 0.630–0.998	(?)	(+) Expert rated	(+) convergent and discriminant	High—strong evidence across properties
Subject understanding interviews [42]	(?)	(?)	(+) *r* = 0.95–0.99	(+) Expert review	(?)	Low—limited evidence
Beranger Framework [43]	(?)	(?)	(+) *k* = 0.81	(+) Validated by psychologists, physicians, and patient association	(?)	Low—limited evidence
Roter Interactional Analysis System (RIAS) [85]	(?)	(?)	(+) *r* = 0.85	(?)	(?)	Very low—minimal evidence

COSMIN Rating System:

(+) Sufficient: adequate psychometric quality (e.g. Cronbach’s *α* ≥ 0.70 for internal consistency; ICC/*r*/*k* ≥ 0.70 for reliability; established content/construct validity through appropriate methods)

(-) Insufficient: below quality thresholds (e.g. *α* < 0.70, ICC/*r*/*k* < 0.70, or inadequate validity evidence)

(?) Indeterminate: property not assessed or insufficient information reported

(±) Inconsistent: conflicting evidence across studies

Overall quality of evidence: reflects the comprehensiveness of psychometric testing, methodological rigour of studies, and consistency of results. High = multiple properties tested with strong methodology; moderate = several properties with good evidence but some gaps; low = limited testing or methodological concerns; very low = minimal or weak evidence

*α* Cronbach’s alpha, *ICC* intraclass correlation coefficient, *r* correlation coefficient, *k* Cohen’s kappa

Although the QuIC did not score as highly on COSMIN criteria as some other tools, it was used more frequently than any other measure across the included studies. It was also the most efficient tool/measure in terms of time and resources, with an average of 7.2 min administration time. The three tools/measures with the highest COSMIN scores and the tool used most frequently were further analysed and compared based on their mode of administration, time required to complete them, and scoring format (Table 4).

**Table 4:** Comparison of administrative properties of identified tools/measures

Tools/measures	Administration mode	Time	Scoring and format
QuIC Questionnaire [18]	Patient self-report	7.2 min	Section A (20 understood/not understood/unsure) Section B (14 Likert questions)
DICCQ [30]	Administered by trained interviewers on a laptop computer in a private consultation room at the field site, where the interviewer read question items aloud to participants in their chosen local language while operating the computer interface, and participants could respond either verbally or by pointing to response symbols on the screen	22.4 min	Section A (9 yes/no) Section B (10 MCQS) Section C (7 completion questions)
PIC measure [166]	Applied by the researcher to the audio-recording/transcript of the recruitment discussion	56 min	Section 2: 25 questions (0–3 points information provision) (0–3 points understanding) Section 3: global judgement
P-QIC [55]	The tool was administered through observation of scripted video vignettes using volunteer actors to depict informed consent encounters; researchers completed the observational tool while viewing these standardised video scenarios	Not given	16 questions (done well, done, done poorly, not done)

## Discussion

This systematic review is the first of its kind to our knowledge and identified a wide range of tools/measures and approaches for measuring participants’ understanding of the IC process in clinical trials. The analysis of 148 studies identified variation in how participants’ understanding is construed, measured, and evaluated across different clinical trial settings. Although participants’ understanding of study information has been established as a prerequisite for conducting ethical clinical trials [174], this review of tools/measures designed to assess such understanding underlines the inconsistency between standardised principles and empirical practices. While many tools/measures aimed to assess ‘understanding’, the conceptual underpinning of this term varied widely, ranging from factual recall to appreciation, reasoning, and voluntariness, which are the elements that align more closely with the decision-making capacity framework [175]. By synthesising evidence on existing tools and their psychometric properties, this review consolidates current knowledge and identifies methodological considerations for improving the assessment of participant understanding in clinical trial consent processes.

The inconsistent evaluation of ‘understanding’ reflects the challenge of translating broad bioethical principles into standardised practice. While this variability may compromise consistency across consent processes, it highlights the multifactorial nature of the construct ‘understanding’ that resists simple standardisation. This raises questions about whether universally applicable assessment tools/measures and approaches are feasible or whether more flexible frameworks are needed. The breadth of constructs labelled as ‘understanding’ (e.g. factual recall, appreciation, reasoning, voluntariness) suggests a lack of shared specification of what should be measured in consent evaluations. One potential response is the development of a core set of domains for consent understanding (and related constructs) that should be assessed across trials, while allowing additional, context-specific domains for particular settings (e.g. early-phase oncology versus public health trials). Such a core domain approach may improve comparability across studies without implying that a single universal measure is appropriate for all contexts. Standardisation should therefore remain context-sensitive and co-produced with patients and the public, balancing comparability with local relevance.

There was also variation in the timing of assessment, with approaches adopted ranging from evaluation immediately after obtaining consent, during the recruitment process, or after a significant time had passed. These practices raise questions about the ideal timepoint to assess comprehension or recall. It presents the need for uniformity across studies and justification in selecting appropriate recall assessment timeframes. Delayed assessment may reflect knowledge retention, but it may not capture any other aspects of understanding the participant had during the recruitment discussions. Factors beyond the initial consent process may have influenced aspects of understanding evaluated at a later time [13, 176], which therefore fail to capture the quality of recruitment discussion or make any claim about participants’ understanding during consent.

Based on the COSMIN criteria, there were only three measures that demonstrated high-quality psychometric properties, and this indicates that there are gaps in the methodological rigour of most of the available tools/measures used in assessing participant understanding. The limited validation of many of these assessment tools also raises concerns regarding the validity and reliability of the data they report [177]. The limited validation of many of these assessment tools also raises concerns regarding the validity and reliability of the data they report [177]. The original QuIC validation relied on limited internal consistency estimates, small test–retest samples, and indirect construct validity without factor analysis or responsiveness testing, which falls short of contemporary criteria such as COSMIN criteria.

Despite being moderate on COSMIN criteria, the QuIC questionnaire was used most frequently across the studies, possibly because it was quick to administer, suggesting that pragmatic considerations (brevity, low burden, ease of scoring, and acceptability in recruitment workflows) may outweigh measurement rigour when tools/measures are selected. This highlights the critical balance between scientific rigour and feasibility in real-world clinical trials research settings. Highly validated tools may be less implementable, whereas more implementable tools may provide weaker inference about comprehension. Future research should explicitly evaluate this trade-off, including implementation outcomes (time, training, acceptability) alongside psychometric properties, and consider whether brief tools can be strengthened through targeted validation and calibration work.

Moreover, among the highest-scoring tools/measures identified in this review, each reflects distinct validation contexts and design assumptions that shape its applicability to real-world consent processes [30, 55, 166]. Although the DICCQ tool has been cited as one of the few empirically validated measures of IC comprehension in low-literacy research settings, it is not yet widely adopted globally. Its published validation and adaptation work has been concentrated in a limited number of settings (notably The Gambia, with later cross-cultural adaptation work reported in rural Kenya) [30, 178]. However, its audio-based administration in local spoken languages, rather than written text, supports the assessment of comprehension for IC in settings where the written format of local languages is limited or absent [179]. For P-QIC, caution is also warranted when interpreting its applicability to real-world recruitment settings, because its evaluation relied on structured video scenarios rather than live consent encounters [55]. Although the PIC tool study [166] was limited in its PRO-EDI reporting, the more recent evaluation of PIC within OPTiMISE [180] purposively sampled recruitment discussions to achieve maximum variation across patient gender, study centre, and recruiter.

The demographic characteristics varied across trials and studies, including age, gender distribution, and education level. This variation underscores the importance of explicitly considering equity and participant characteristics in the development and validation of IC assessment tools [9, 100, 181], so that the populations for whom these tools are intended, and to whom they may be reasonably applied, are transparent. However, limited reporting of demographic characteristics meant that it was not possible to examine associations meaningfully between levels of understanding and different participant groups. While incorporating diverse perspectives during tool development is necessary, it is unlikely that we can develop something that is applicable across all settings; context-specific validation, and in some cases alternative or tailored assessment approaches (e.g. for populations with low literacy), may still be required. Furthermore, the majority of the tools were developed and applied in the USA or UK, which may limit their generalisability and transferability to other healthcare systems and cultural contexts [182].

Limited PPIE input was also observed across the tool/measure development, with only a few studies reporting any form of patient or public involvement. Even where PPIE was included, its scope was restricted to specific stages such as feedback on drafts rather than comprehensive involvement or co-production throughout instrument development. This poses a missed opportunity, as meaningful PPIE integration could enhance the relevance, acceptability, and effectiveness of assessment tools [183, 184]. We recommend that future tools/measures when developed incorporate PPIE from the start, i.e. when identifying and defining constructs and priority domains, to ensure relevance to participants’ lived experiences [185]. Evidence from instrument development literature shows that limiting PPIE to late-stage feedback is insufficient; instead, PPIE contributors should be meaningfully involved throughout the full lifecycle of tools/measures development (from construct definition and item generation to piloting, validation, and dissemination) to ensure relevance, comprehensibility, and legitimacy of the resulting tool [186]. Reporting should also describe who was involved, including demographic and experiential diversity, to allow readers to judge inclusivity and avoid tokenistic involvement [187, 188].

This review identifies the need for a better, well-validated tool/measure to assess participant understanding during recruitment discussions or further validation of existing tools/measures. It should ideally also enable real-time assessment during recruitment discussions, hence minimising the limitations of delayed evaluation. It should be tested for its reliability and validity, and should have PPIE incorporated throughout its development phase. For practical implementation, it should be relatively quick to implement and applicable across various contexts and research settings. In practice, research ethics committees (RECs) may rely largely on investigators’ descriptions of proposed measures. It is therefore important that investigators routinely provide RECs with clear information on the validity and reliability of tools used to assess participant understanding. Also, given the importance of feasibility, investigators should consider the practical feasibility and appropriateness of these tools within their specific research contexts and share those with the REC. While standardised tools for assessing participant understanding may offer value, their routine use in all trials could impose an additional burden on investigators and participants. Therefore, a tool/measure or approach that can be used during recruitment discussions would be more feasible. These tools may be helpful for research purposes, training recruiters in effective recruitment discussions, evaluating comprehension of consent materials, and identifying sections of information sheets that are difficult to understand and require revision.

This SR had several limitations. Firstly, the inclusion of only English-language publications, which is a substantial limitation in a field where consent comprehension is shaped by language, culture, and local research infrastructures. As a result, our findings are likely skewed toward tools developed and used in Western/English-language contexts, and we may have missed relevant measures developed, adapted, or validated in other languages. Secondly, excluding grey literature might have omitted valuable unpublished assessment approaches. Finally, the heterogeneity of reporting across studies limited our ability to conduct a more detailed comparison of tool effectiveness across different demographic contexts. It also prevented a definitive conclusion about which single tool/measure or approach is ‘best’. Rather, the findings helped identify which tools have the strongest psychometric evidence to date and where feasibility–rigour trade-offs likely influence uptake.

## Conclusion

Across 148 included studies, we found substantial heterogeneity in how “understanding” was conceptualised and assessed, and only three tools (DICCQ, PIC, P-QIC) demonstrated high-quality psychometric properties using COSMIN criteria. In contrast, the most frequently used tool (QuIC) demonstrated only moderate quality, suggesting that feasibility and implementability may drive tool choice more than measurement robustness. Future work should prioritise (i) rigorous development and validation of measures, (ii) context-sensitive standardisation of what is assessed and when, and (iii) meaningful patient and public involvement across the full tool development lifecycle. Addressing these gaps will allow researchers to build more effective methods to ensure the plurality of ways that participants understand are assessed, thereby strengthening the implementation of ethical clinical research. Improved understanding of consent can enhance patient outcomes, satisfaction, and trust, crucially impacting clinical practice and patient care.

## Supplementary Information

Supplementary Material 1.

Supplementary Material 2.

Supplementary Material 3.

Supplementary Material 4.

## Data Availability

The datasets used and/or analysed during the current study are available from the corresponding author on reasonable request.

## Abbreviations

BICEP: Brief Informed Consent Evaluation Protocol
COSMIN: COnsensus-based Standards for the selection of health Measurement INstruments
CINAHL: Cumulative Index to Nursing and Allied Health Literature
DICCQ: Deaconess Informed Consent Comprehension Questionnaire
DICCT: Digitised Informed Consent Comprehension Test
eIC: Electronic Informed Consent
IC: Informed consent
ICQ-4: Informed Consent Questionnaire
MacCAT-CR: MacArthur Competency Assessment Tool for Clinical Research
MeSH: Medical Subject Headings
PIC: Participatory and Informed Consent
PPIE: Patient and Public Involvement and Engagement
PRISMA: Preferred Reporting Items for Systematic Reviews and Meta-Analyses
P-QIC: Process and Quality of Informed Consent
QuIC: Quality of Informed Consent
RIAS: Roter Interactional Analysis System
SR: Systematic review
UTD: Understanding Treatment Disclosures
