# Supplementary material for: Measuring and evaluating participant understanding of consent processes in clinical trials: a systematic review
Source: Trials. 2026 Mar 4;27:192. doi: 10.1186/s13063-026-09582-x (PMC12964858; doi:10.1186/s13063-026-09582-x)
Supplement: Supplementary file 1 — Supplementary Material 1. [file 13063_2026_9582_MOESM1_ESM.docx]

**Supplementary Table 1: MEDLINE Search Strategy**

Database(s): Ovid MEDLINE(R) 1946 to present
Search Strategy: 13.03.2023

| **#** | **Searches** | **Results** |
| --- | --- | --- |
| **1** | **Informed Consent/** | **38619** |
| **2** | **Third-Party Consent/** | **3786** |
| **3** | **Consent Forms/** | **1736** |
| **4** | **consent.ti. or consent.ab. /freq=3** | **16486** |
| **5** | **or/1-4** | **45169** |
| **6** | **(trials or ((clinical or medical) adj research)).mp.** | **969672** |
| **7** | **(patient* or participant* or research subject? or volunteer*).mp.** | **9026517** |
| **8** | **(acceptance or advocacy or autonomy or communication or comprehension or education or knowledge or literacy or understanding).mp.** | **3257358** |
| **9** | **(quality or standards or standardi#ed).mp.** | **2421077** |
| **10** | **8 or 9** | **5187019** |
| **11** | **5 and 6 and 7 and 10** | **3031** |
| **12** | **limit 11 to english language** | **2832** |
