# Supplementary material for: Measuring and evaluating participant understanding of consent processes in clinical trials: a systematic review
Source: Trials. 2026 Mar 4;27:192. doi: 10.1186/s13063-026-09582-x (PMC12964858; doi:10.1186/s13063-026-09582-x)
Supplement: Supplementary file 2 — Supplementary Material 2. [file 13063_2026_9582_MOESM2_ESM.docx]

***Supplementary Table 2: Participant and tool characteristics***

| **#** | **Author Year** | **Age (years)** | **Sex/Gender** | **Level of Education** | **Tool name** | **Related Domains (Verbatim from included study)** |
| --- | --- | --- | --- | --- | --- | --- |
| 1 | Abd-Elsayed et al. 2012 (28) | Mean: 62 (SD = 13) | Male: 68% Female: 32% | High School 39%; Undergraduate 45%; Graduate 16% | Patient Survey | understanding of the study intervention and major associated risk, the relationship between patients’ demographic and clinical characteristics, their personal belief about risk of participation in research, presence of friends or family members during the interview, time to make the decision, and whether patients felt pressured to participate. |
| 2 | Addissie et al. 2016 (29) | Mean: 27.3 (SD = 6.3) | Female: 100% | unable to read & write 58 (29.1%); Able to read & write 17 (8.5%); Primary school 50 (25.1%); Secondary school 51 (25.6%); Diploma &above 23 (11.6%) | Modular Informed Consent Comprehension Assessment (MICCA) Brief Investigator Questionnaire (BIQ) Quality of Informed Consent (QuIC) process assessment tools | Comprehension was assessed as a function of understanding and recall.  Disease information [Q302] Information about the study[Q201, 203, 204, 208] Aim /purpose of the study[Q308A, 308B] Selection criteria [Q301] Procedure of sample collection [Q310A, 310C] Tasks to be performed by participants [Q311A] Risk of the study/ side effect of sample collection [Q312A, 312B] Benefit of the study [Q206, 207,209, 309C] Confidentiality [Q205, 213] Participant right [Q202, 210, 212, 304, 305, 306] |
| 3 | Afolabi et al. 2014 (30) | Mean: 37.06 (SD = 15) | Men: 91 (36.8%) Women: 156 (63.2%) | Western education 62 (25.1%); no western education 185 (74.9%) | Digitised Informed Consent Comprehension Questionnaire (DICCQ) | voluntary participation, rights of withdrawal, study knowledge, study procedures, study purpose, blinding, confidentiality, compensation, randomisation, autonomy, meaning of giving consent, benefits, risks/adverse effects, therapeutic misconception and placebo. |
| 4 | Afolabi et al. 2015 (31) | **Intervention:** 18–25 : 14.8% 26–33 : 32.3% 34–41 : 25.8% 42–49 : 18.1% 49+ : 9.0% **Control:** 18–25 : 22.4% 26–33 : 28.2% 34–41 : 22.4% 42–49 : 21.8% 49+ : 5.1% | **Intervention:** Male: 38.1% Female: 61.9% **Control:** Male: 35.9% Female: 64.1% | **Intervention:** Formal: 26.5% No formal: 73.5% **Control:** Formal: 18.6% No formal: 81.4% | Multimedia informed consent tool and a computerized, audio questionnaire | Recall and understanding by use of a multimedia informed consent tool |
| 5 | Ahalt et al. 2017 (32) | Mean: 59 | Not reported | Not reported | “teach-to-goal” informed consent comprehension assessment tool. | study’s purpose, eligibility criteria, study procedures, the risks associated with participation, and participants’ rights |
| 6 | Alexa-Stratulat et al. 2018 (33) | **Phase I:** 18–25 (66%) [no info on remaining 34%] **Phase II/III:** 46–60 (50%) [no info on remaining 50%] | **Phase I only** Male: 72% Female: 28% | **Phase I:** Finished high school: 76%  **Phase II/II:** Finished high school: 46% | Modified Quality of Informed Consent (QuIC) questionnaire | Objective understanding (Q1-Q20) (agreement to participante in RCT, Purpose of research, risks and benefits, side effect, aims of researcher, randomisation, confidentiality, possibility to withdraw, who to contact, pay for medical care in the event of side effects, amount of time to be contributed by participants, other available treatment option)  Subjective understanding (Q21-Q34) (study type, duration, purpose of research, risks and benefits, understanding of the procedures and treatments, confidentiality, who to contact, other available treatment option, voluntariness) Factors influencing subjective and objective understanding (age, gender, education, previous participation) |
| 7 | Allen at al. 2017 (34) | Mean: 43.8 (SD = 9.3) | Male: 85% Female: 15% | Average: 11.5 years (SD = 2.01) | Consent quiz (CQ) instrument | risks and potential benefits of study participation, the voluntary nature of and alternatives to participation, and study procedures |
| 8 | Apseloff et al. 2013 (35) | Not reported | Male: 100% | Not reported | Questionnaire to assess comprehension of the consent form and demographic information​ | Not reported |
| 9 | Arora et al. 2011 (36) | Mean: 25.5 (SD = 6.2) | Male: 100% | primary education or above: 96%; high school education: 48% | Informed consent comprehension questionnaire | Background (6 items) Design (9 items) Rights (12 items) Miscellaneous (6 items) |
| 10 | Asher et al. 2022 (37) | Mean: 58.3 (SD = 12.8) | Male: 90 (45.9%) Female: 106 (54.1%) | Elementary/Junior high: 7.6%; Professional school /agricultural: 11.7%; High school: 11.2%; Yeshiva (religious college): 2.5%; Non-academic training: 23.4%; Academic: 43.7%. | hospital-approved anonymous questionnaire for evaluating their attitudes towards clinical trials | knowledge (placebo, standard of care, signing informed consent, refusal from the study, choice of experimental drug, benefits to recruiter) |
| 11 | Atal et al. 2018 (38) | Mean: 32.40 (SD = 4.08) Range: 24–41 | Female: 100% | Leaving certificate: 5; Bachelor’s degree: 8; Master’s degree: 2 | Modified Quality of Informed Consent (QuIC) questionnaire | "Objective understanding" and "Subjective understanding" of informed consent |
| 12 | Ballard et al. 2020 (39) | **Survey participants:** Under 20: 18 (1.6%) 20–30: 79 (6.8%) 31–40: 232 (20%) 41–50: 213 (18.4%) 51–60: 183 (15.8%) 60+ : 403 (34.7%)  **Interview participants** 20–30: 2 (9%) 31–40: 6 (26%) 41–50: 7 (30%) 51–60: 8 (35%) | **Survey Participants:**  Male: 487 (42%) Female: 640 (55.2%) Other: 7 (0.6%)  **Interview Participants**:  Male: 7 (30%) Female: 16 (70%) | **Survey Participants:** No schooling: 30 (2.6%) Primary: 50 (4.3%)  GCSE or equivalent: 372 (32.1%) A level or equivalent: 249 (21.5%) Bachelor’s: 215 (18.5%) Master’s: 85 (7.3%) Doctorate: 21 (1.8%) Other: 70 (6%)  **Interview Participants:** GCSE or equivalent: 4 A level or equivalent: 5 Bachelor’s: 11 Master’s: 1 | survey + indepth interview | For qualitative part, themes explored are: “I don’t remember, maybe I didn’t understand it  “I don’t remember much, and I don’t understand everything, but that’s OK”.completely”. |
| 13 | Barrett 2005 (40) | Mean: 64.3 (SD = 11.9) Range: 39 – 76 | Male: 75% Female: 25% | Some high school: 25%; High school graduate or GED: 50%; Graduate or professional school: 25% | Quality of Informed Consent (QuIC) questionnaire | objective and subjective understanding Perception of informd consent process |
| 14 | Behrendt et al. 2011 (41) | Mean: 39.6 Range: 18–69 | Male: 6 Female: 4 | Not reported | half-standardised interview guideline | Understanding, satisfaction, and needs of the patients |
| 15 | Benson et al. 1985 (42) | **Depression study:** Mean: 65 (Range: 59–71) **Schizophrenia study:** Mean: 37 (Range: 21–55) | **Depression study:** Male: 87% Female: 13%   **Schizophrenia study:** Male: 100% | **Depression study:** Less than a full high school education: 9 (37%) High school graduate: 5 (21%) Some college: 6 (25%) College degree: 4 (17%)  **Schizophrenia study:** Less than a full high school education: 4 (17%) High school graduate: 9 (37%) Some college: 9 (37%) College degree: 2 (8%) | Systematic observation using audio and videotape, standardized interaction rating forms, and subject understanding interviews. | Understanding of the research project’s nature and purpose, as well as its risks, benefits, alternatives and consequences |
| 16 | Beranger et al. 2019 (43) | Not reported | Not reported | Not reported | Framework used for the interview to assess the parents’ and child’s understanding of the consent information | Understanding of nine elements of informed consent, including participation, purpose, design, duration, individual benefits, collective benefits, right to withdraw, risks, and alternative options. |
| 18 | Bergenmar et al. 2011 (44) | Mean: 60.0 (SD = 10.8) | Men: 124 (46%) Women: 144 (54%) | Academic education: 47% | Quality of Informed Consent (QuIC) | Knowledge about randomization, benefits for future patients, participation in a research trial, and the right to withdraw |
| 17 | Bergenmar et al. 2008 (45) | <45: (9.21%), 45–64: (50.35%), >65: (40.42%) | Men: 135 (48%) Women: 147 (52%) | Compulsory school (1–9 years): 24%, Senior high school (10–12 years): 29%, University education (13–16 years): 38%, Higher university education (>16 years): 9% | Quality of Informed Consent (QuIC) questionnaire | Research purpose and procedures, duration, experimental procedures, Potential risks or discomforts, Benefits to self and others, Alternatives to participation, Confidentiality, Procedures in the event of research injury, Study contacts, Voluntary nature of participation. |
| 19 | Bhansali et al. 2009 (46) | Mean: 51.6 (SD = 9.9) | Male: 83.4% Female: 16.6% | Uneducated: 5 (11.3%) Primary level: 14 (27.7%) Matriculate level: 7 (15.9%) Graduate: 12 (27.7%) Postgraduate: 5 (11.3%) Doctorate: 1 (2.2%) | Informed Consent comprehension questionnaire | Background details (why the study is conducted, by whom, involved centers, reasons for participation, study conduct) Design (number of groups, determinants of group allocation, knowledge of treatment, drug administration, follow-up visits) Rights (withdrawal, refusal, compensation, confidentiality) Miscellaneous (study approval, sponsors, involved centers, contact information, post-study treatment) |
| 20 | Boyd et al. 2021 (47) | Not reported | Male: 1 (father) Female: 9 (7 mothers, 2 grandmothers) | Not reported | Deaconess Informed Consent Comprehension Test (DICCT) for the initial closed questions, and a semi-structured interview schedule for open-ended questions. | Comprehension of the clinical trial’s purpose, understanding participant information sheets and consent forms, motivations for enrolling their child, and the importance placed on the informed consent process. |
| 21 | Brandberg et al. 2016 (48) | Mean: 55 (SD = 10.6) Distribution: <45: (16%), 45–64: (68%), ≥65: (16%) | Male: 22 (18%) Female: 103 (82%) | Compulsory school (1–9 years): 14%, Senior high school (10–12 years): 16%, University education (13–16 years): 11%, Higher university education (>16 years): 39%, Data missing: 20% | Quality of Informed Consent (QuIC) questionnaire | Knowledge about randomization, benefits for future patients, participation in a research trial, and the right to withdraw. |
| 22 | Campbell et al. 2008 (49) | **Control group:** 20–29 (4.9%), 30–39 (7.4%), 40–49 (17.3%), 50–59 (28.4%), 60–69 (12.3%), 70–79 (13.6%), ≥80 (4.9%) **Intervention group**: 20–29 (8.1%), 30–39 (12.9%), 40–49 (16.1%), 50–59 (17.7%), 60–69 (25.8%), 70–79 (12.9%), ≥80 (3.2%) | **Control group:** Male: 54.3% Female: 38.3%   **Intervention group:**  Male: 61.3% Female: 35.5% | Not reported | Knowledge of Clinical Trials | Knowledge of clinical trials, perceptions about clinical trials, likelihood of participation in clinical trials |
| 23 | Campbell et al. 2017 (50) | Younger group: 20–39 (63.8%) Older group: 40–59 (36.2%) | Male: 89.6% Female: 10.4% | Primary (Grade 7 or less): 31.8%, Secondary (Grade 8 or more): 68.2% | University of California, San Diego Brief Assessment of Capacity to Consent Questionnaire (UBACC), | purpose of the study, protocol procedures, study risks and benefits, and the voluntary nature of participation |
| 24 | Cervo et al. 2013 (51) | Median: 56 Range: 18–84 | Male: 86 Female: 344 | Elementary school: 22.7%; Middle school: 29.7%; High school/University: 47.5% | CRO-Biobank questionnaire for patients | The questionnaire investigated patients’ knowledge of biobanks before and after participating in the study, the perceived quality of our informational methods, and how well the patients felt their privacy had been respected |
| 25 | Chaisson et al. 2011 (52) | Median: 33 Distribution: <20: (0.4%), 20–29: (32%), 30–39: (45%), 40–49: (18%), 50–59: (3%), 60–69: (<1%), ≥70: (<1%) | Male: 28% Female: 72% | primary: 28%; secondary: 56%; tertiary: 9%; and no formal education: 6% (table info conflicts with descriptive data) | enrollment quiz (Quiz1) | Study purpose, Placebo, Blinding, Procedure, Voluntariness, Adherence, Risk, Compensation, Background. |
| 26 | Chapman et al. 2015 (53) | Median: 27 IQR: 24–42 | Male: 76% Female: 24% | <8th grade: 33%; >8–12th grade: 44%; >12th grade: 17% | informed consent assessment tool (ICAT) | Questionnaire items included inquiries about site staff who administered the consent process, the use of interpreters, time spent administering consent, the number of visits to complete the consent process, and the approaches for obtaining consent from persons with low literacy. Additional items addressed approaches to assess participant understanding such as the use of short forms or translated forms, the use of visual aids, the presence or absence of family and friends during the consent process, and protocols related to a participant signing the form. Suggestions for improving the informed consent process were also solicited. |
| 27 | Chappuy et al 2010 (54) | Not reported | **First interview:** 14 fathers 37 mothers  **Second interview:**  10 fathers 29 mothers | Not reported | Questions asked during the interview addressing the level of understanding | Participation in a research protocol Aim of the protocol Course of the protocol Principle of randomization Individual benefit Collective benefit Risks Alternatives Voluntary nature of participation Duration of participation Freedom to withdraw from the project at any time |
| 28 | Choi et al 2019 (11) | 30–39: 4 (19.0%) 40–49: 4 (19.0%) 50–59: 9 (42.9%) 60–69: 3 (14.3%) ≥70: 1 (4.8%) | Men: 10 (47.6%) Women: 11 (52.4%) | Elementary school or below: 2 (9.5%) Middle school: 2 (9.5%) High school: 8 (38.1%) University or above: 9 (42.9%) | Interview Guide: English | understanding (Voluntary participation and withdrawal Random allocation Alternative procedure Clinical trial phase Potential risks Period and method of treatment Degree of understanding about written information Willingness to use written information) |
| 29 | Cohn 2009 (55) | Not reported | Not reported | Not Reported | Process-Quality of Informed Consent (P-QIC) | (a) an accurate and complete description of the elements necessary for informed consent for clinical research, (b) the essential elements identified for effective patient-provider communication, and (c) items identified in the literature as critical to comprehension. |
| 30 | Collins et al. 2023 (56) | **Research participants**: Mean: 59.1 (SD = 11.8) **Routine care patients**: Mean: 59.8 (SD = 13.5) | **Research participants:**  Male: 31% Female: 69%   **Routine care patients:** Male: 28% Female: 72% | Not reported | English Questionnaire (by J. Collins) | Risks and benefits of interventions Availability of alternative options Personal opinion in the final decision Confidence in receiving the best management Awareness of research participation |
| 31 | Corneli et al. 2012 (57) | **Group 1**: Mean: 23.2 **Group 2**: Mean: 23.7 **Group 3**: Mean: 23.3 | Female: 100% | Average number of years in school **Group 1:** 6.7 years **Group 2:** 6.8 years **Group 3:** 7.5 years | QuIC questionnaire | Eligibility criteria ARV intervention Nutrition intervention Blood draws Breastfeeding protocol |
| 32 | Criscione et al. 2003 (58) | Mean: 44.9 (SD = 9.8) | Male: 20% Female: 80% | Median: 12.5 years | consent process questionnaire (CPQ) | Not reported |
| 33 | da Fonseca et al. 1999 (59) | Not reported | Male: 100% | Basic education: 24%; High school education: 47%; University education: 28% | Questions to construct the vaccine knowledge score | randomization, blinding, Compensation and Reimbursement, Behavioral Requirements, Vaccine Effects on Testing, Eligibility for Future Vaccines, Potential Side Effects, Immune Response and Infection Risk |
| 34 | Das et al. 2014 (60) | Median: 27 (Range: 18–51) | Male: 88% Female: 12% | Cannot read or write: 2 (12%) Cannot read but can write name: 11 (69%) Primary schooling: 3 (19%) | Semi-structured interview guide | Perception of research, Voluntariness, Factors influencing participation, Concept of being informed. |
| 35 | Davis et al. 1998 (61) | Median: 48 (Range: 19–85) | Male: 44 (24%) Female: 139 (76%) | average: 11.9 years | structured oral questionnaire | Not reported |
| 36 | De Oliveira et al. 2017 (62) | Median: 26 (IQR: 22, 33) | Male: 132 (61%) Female: 84 (39%) | Not reported | Computer-based questionnaire | Not reported |
| 37 | Dellson et al. 2019 (63) | Patient: Median: 62 (Range: 21–76) Physician: Median: 51 (Range: 32–65) | **Patients:**  Male: 9 (19.6%) Female: 37 (80.4%)   **Physicians:**  Male: 12 (70.6%) Female: 5 (29.4%) | **patient's eduction:**  Compulsory school (1–9 years): 7 (15.2%) Senior high school or vocational training (10–12 years): 16 (34.8%) College or university (13–16 years): 23 (50.0%)  **physician's education**: Not reported | perceived understanding, seven mirroring questions (7Q-PAT); and Patient Understanding of Research (Q-PUR) questionnaire | Understanding of oral information, understanding of written information, understanding of words and expressions used, understanding of treatment and follow-up, understanding of side effects, understanding of the purpose of the research study. |
| 38 | Diemert et al. 2017 (64) | Mean: 29.3 (SD = 8.9) Range: 18–50 | Female: 46.7% | primary education: 37 (35.2%) secondary education: 33 (31.4%) post-secondary education: 25 (23.8%) post-graduate education: 6 (5.7%) illiterate: 4 (3.8%) | A semi-structured questionnaire consisting of 32 questions. | Knowledge of the purpose of the clinical trial, the study methods, the duration of the trial, the participants’ rights, and the potential risks and benefits of participation. |
| 39 | Ditae et al. 2018 (65) | Mean: 24.5 (SD = 5.7) Range: 15–36 | Female: 100% | No formal education: 1 (3.3%) Did not complete primary education: 14 (46.7%) Primary Leaving Education: 6 (20%) Ordinary level education (Uganda Certificate of Education - UCE): 7 (23.3%) Advanced level education or above: 2 (6.7%) | modified version of the Quality of Informed Consent (QuIC) and semi-structured interview | A1. Follow-up duration after birth:  A2. Number of villages participating:  A3. Action if hand gel runs out:  A4. Post-birth action:  A5. Handgel contents:   B1. Study goals, B2. Study duration, B3. Treatments/procedures, B4. Alternatives, B5. Contact for concerns, B6. Voluntariness, B7. Understanding at consent  C1. Researcher-read, C2. Slideshow, C3. Video C4. Preferred consent model and reason: [Input] |
| 40 | Dresden et al. 2001 (66) | Mean: 39.4 (SD = 12.1) Range: 18–80 | Female: 52% | Middle school: 4%; High school: 62%; College: 29%; Graduate school: 5% | Postconsent test | Questions 1 (purpose),  2 (inclusion/exclusion criteria), 3 (randomization),  4 (length),  5 (risks/side effects),  6 (benefits),  7 (alternative treatments), 8 (compensation for injury), 9 (confidentiality), 11 (voluntary participation) and 12 (withdrawal without penalty |
| 41 | Duvall Antonacopoulos et al. 2016 (67) | Mean: 34.72 (SD = 11.81) Range: 19–73 | Men: 127 Women: 108 Other: 1 | at least one college or university degree: 113 (48.1%) | 10-item comprehension quiz | the purpose of the informed consent, whom to contact for questions and/or concerns, the purpose of the study, the study task requirements, potential risks/discomfort, anonymity/confidentiality, and the right to withdraw from the study. |
| 42 | Eichner et al. 2020 (68) | Mean: 65 (SD = 12.1) | Female: 38.1% | Primary/secondary education: 60.4%; Middle school: 21.6%; A-levels: 18.0% | A 20-item questionnaire. | Diagnosis & Prognosis Data Protection Patient Rights Study Methodology Patient's involvement Study Conduct Study Risks & Benefits Study Content |
| 43 | Falvo et al. 2021 (69) | Mean: 71 (SD = 9.3) Range: 45–86 | Men: 11 Women: 11 | Primary: 1; Secondary: 11; University: 9 | Interview grid | meaning participants attributed to the informed consent process, their perceived barriers to accessing and understanding the information sheet and declaration of consent form, and their preferences regarding the document’s format and content, including their opinion on using visual aids such as a video to support the informed consent process. |
| 44 | Ford et al. 2008 (70) | Mean: 71 (SD = 8.6) | Male: 73 (53.7%) Female: 64 (46.3%) | High school diploma or less: 39 (28.7%) Some college or trade school: 50 (36.8%) College degree: 47 (34.6%) | Quality of Informed Consent Questionnaire (QuIC) (part A). | Not Reported |
| 45 | Fortney et al. 1999 (71) | More than half participants range: 18–25 | Female: 100% | 12 years of schooling: **African site:** 22% **US site**: 91% | interview forms: (a) for early contact (b) for recal | interviewers asked each participant the purpose of the study; why she agreed to participate; whether she knew what study procedures would be used; how often she needed to return to the clinic; how much she would be paid; whether she thought that the reimbursement was an appropriate amount; whether she was offered other contraceptive methods; whether she felt free to withdraw from the study; who could look at her medical records; whether she cared who looked at her records. |
| 46 | Freeman et al. 2013 (72) | **MacLiver Group:** Mean: 39 (SD = 10.6) | **MacLiver Group**:  Male: 48% Female: 52% | **MacLiver Group:** High school or less: 24% Technical school: 22% Associate/Bachelor degree: 39% Post-college graduate degree: 7% Unknown/missing: 8% | MacArthur Competence Assessment Tool for Clinical Research (MacCAT-CR), adapted for the LD setting as MacLiver | understanding the donor evaluation process, understanding the risks and benefits of donation, understanding the right to refuse without consequence, appreciating the impact of donation, expressing a clear decision to move forward or not, and providing clear reasoning for that decision. |
| 47 | Gad et al. 2022 (73) | Median: 63 [IQR: 54, 71] | Male: 114 (50%) Female: 115 (50%) | Short education: 22% (50) Medium education: 13% (29) Long education: 65% (148) | Quality of Informed Consent (QuIC), part A, | A: Alternatives to participation B: Potential risks or discomforts C: Research purpose D: Benefits to self and other E: Confidentiality F: Voluntary nature of participation G: Study contacts |
| 48 | Gammelgaard et al. 2004 (74) | **Participants:** Mean: 60 **Non-participants:** Mean: 61 | **Participants:** Male: 75% Female: 25%   **Non-participants:**  Male: 70% Female: 30% | Not reported | questionnaire (A) and (B) | Patients’ recollection of information about the trial Participant perception Reason for participating Reason for not participating |
| 49 | Gertsmen et al. 2020 (75) | <16: 1 (2%) 20–29: 10 (24%) 30–39: 23 (56%) 40–49: 5 (12%) 50–59: 2 (5%) ≥60: 0 (0%) | Not reported | Some high school, no diploma: 2 (5%) High school graduate: 8 (20%) Some postsecondary studies, no degree: 6 (15%) College or vocational degree: 10 (24%) Undergraduate degree: 7 (17%) Postgraduate degree: 7 (17%) Other: 1 (2%) | STRIPES Questionnaire | Topic of study (1) Eligibility (2) Placebo/randomization (3) Risks: relative (4A) Risks: specific (4B) Voluntary participation (5) Privacy/confidentiality (6) |
| 50 | Ghormley et al. 2011 (76) | **Control**: Mean: 37.56 (SD = 9.99) **Unimpaired-depressed:** Mean: 35.75 (SD = 10.95) **Impaired-depressed**: Mean: 40.57 (SD = 11.99) | **Control:**  Male: 6 Female: 10  **Unimpaired-depressed:**  Male: 5 Female: 19  **Impaired-depressed:**  Male: 2 Female: 10 | **Control:** 16.00 years (SD = 2.45) **Unimpaired-depressed:** 12.94 years (SD = 1.88) **Impaired-depressed:** 11.86 years (SD = 1.96) | the Understanding Treatment Disclosures Scale (UTD) | 1) depression and its symptoms, 2) a proposed treatment, 3) symptoms which the treatment is expected to relieve and the likelihood this will occur, 4) potential risks and the likelihood they will occur, and 5) a description of alternative treatments and their potential risks and benefits |
| 51 | Gillespie 2017 (77) | **Pre intervention group**: Mean: 66.67 (SD = 17.35) **Post intervention group**: Mean: 75.80 (SD = 8.70) | **Pre-intervention group**:  Male: 67% Female: 33%   **Post-intervention group**:  Male: 100% | **pre intervention group:** 0-8 years: 11%; 9-12 years: 17%; High School/GED: 22%; 1-2 yrs college: 17%; 4 yrs college: 33%  **post intervention group:** 0-8 years: 0%; 9-12 years: 40%; High School/GED: 0%; 1-2 yrs college: 0%; 4 yrs college: 60% | QuIC questionnaire | objective and subjective understanding |
| 52 | Goldberger et al. 2011 (78) | Mean: 61 (SD = 16) | Male: 45 Female: 18 | Highest education, college or above: Oral: 64% Written: 73% Video: 50% | Questionnaire to assess patient comprehension Questionnaire to assess patient satisfaction | understanding of the procedure and its potential complications. |
| 53 | Golembiewsky et al. 2021 (79) | Mean: 45.5 (SD = 16.0) | Male: 232 (31.6%) Female: 502 (68.4%) | Less than high school: 72 (9.8%) High school graduate or GED: 249 (33.9%) Some college: 234 (31.9%) Bachelor’s degree: 106 (14.4%) Master’s, professional, or doctorate: 73 (9.9%) | Quality of Informed Consent (QuIC) scale - Subjective Understanding subscale; 6 statements developed by the research team that reflected factual information present in all 3 e-consent versions; Decision-Making Control Instrument | Not Reported |
| 54 | Gota et al. 2018 (80) | Mean: 44 (SD = 14.1) Range: 18–74 Median: 45 | Male: 120 Female: 80 | Illiterate: 26; Up to secondary school: 90; College: 84 | Quality of Informed Consent (QuIC) | Level of interest shown by participant Knowledge of participant pertaining to the trial People involved in participants' decision to enrol Factors encouraging participation |
| 55 | Griffin et al. 2006 (81) | <60: 414 (23.1%) 60–69: 947 (52.9%) ≥70: 428 (23.9%) | Male: 100% | <High school: 406 (32.0%) ≥High school: 868 (68.0%) | Three multiple-choice questions | Study purpose, study medication name, main side effect of medication |
| 57 | Guarino et al. 2006 (82) | Mean: 40.7 | Male: 85% Female: 15% | Mean = 14.1 years | Informed Consent Questionnaire (ICQ), reduced to ICQ-4 | Understood their rights as a research participant Knew they would continue to receive medical care Understand the study when consenting Was treatment more difficult than expected Felt potential benefits were explained Felt potential risks were explained Did study meet expectations Would participate in this study if it was repeated Would participate in a VA study again Felt personnel were available to answer questions​​. |
| 56 | Guarino et al. 2006 (83) | Mean: 40.7 Range: 27–72 | Male: 85% Female: 15% | Mean = 14.1 years (SD = 1.9) | Informed Consent Questionnaire-4 (ICQ-4), | "Understand the study when consenting" "Felt potential benefits were explained" "Felt potential risks were explained" "Did study meet expectations" |
| 58 | Harrison et al. 1995 (84) | Not reported | Male: 100% | Not reported | 17 item true false questionnaire | key definitions, possible side effects, possible negative social consequences, unrealistic expectations |
| 59 | Hlubocky et al. 2018 (85) | Median: 60 Range: 33–83 | Male: 45 (45%) Female: 56 (55%) | Some high school: 27%; High school: 28%; College: 20%; Professional: 19%; Some postgraduate: 1%; Other: 5% | Roter Interactional Analysis System (RIAS) | recall (phase I trial research purpose, alternatives to participation, expectations of benefit, and prognosis. Sociodemographic information was obtained.) |
| 60 | Hoffner et al. 2012 (86) | Mean: 56 (SD = 11) | Male: 57.1% Female: 42.9% | Unknown: 2.6%; Some college or less: 41.55%; College graduate or advanced degree: 55.8% | Quality of Informed Consent (QuIC) | objective and subjective understanding |
| 61 | Hofmeijer et al. 2007 (87) | **HAMLET patients**: Mean: 48 (SD = 8) **PAIS patients**: Mean: 69 (SD = 13) | Not reported | Not reported | Recall of trial details | Not reported |
| 62 | Howard et al. 1981 (88) | 30–39: 7 (10.9%) 40–49: 15 (23.4%) 50–59: 25 (39.1%) 60–69: 17 (26.6%) | Male: 56 (87.5%) Female: 8 (12.5%) | <7 years: 1 (1.6%) 7-9 years: 9 (14.1%) 10-11 years: 9 (14.1%) H.S. diploma: 17 (26.6%) Some college: 13 (20.3%) College degree: 8 (12.5%) Advanced degree: 7 (10.9%) | interview guide | Awareness of purpose Awareness of side effects Awareness of the assignment process Awareness of the double blind Comprehensive awareness (To measure the patients" overall comprehension of the trial, every respondent was given one point for each basic component of information that was understood.) |
| 63 | Hu et al. 2022 (89) | Mean: 33 (SD = 11) | Male: 264 (48.3%) Female: 283 (51.7%) | Less than high school 36 (6.6%) High school 148 (27.1%) College 269 (49.2%) Postgraduate 94 (17.2%) | the knowledge survey (8-item survey section) The Electronic Informed Consent Attitudes Scale (eIC Attitudes Scale) | Questions included statements about the concept, content, form, and legal effectiveness of eIC (reliability of eIC, diversity of forms of eIC, consistency of content with paper-based informed consent, diversity of communication methods, use of electronic signatures, methods of information presentation, location, flexibility of eIC, and the requirement for ethics review) |
| 64 | Hughes et al. 2017 (90) | Mean: 35 (SD = 14.05) Range: 18–74 | Male: 65 (32.3%) Female: 136 (67.7%) | Secondary school: 10 (4.8%) Some college: 31 (14.8%) Bachelor's degree: 50 (23.8%) Master's degree: 58 (27.6%) Doctoral degree: 44 (21.0%) Other: 17 (8.1%) | 15-item placebo knowledge questionnaire | side effects and effectiveness of placebo |
| 65 | Hutchison et al. 2007 (91) | Overall: Mean: 57 Range: 38–76 Patients (no previous RCT): Mean: 63 Range: 43–81 Research nurses: Mean: 41 Range: 28–57 | **Patients (previous RCT):**  Male: 10 Female: 16 Patients   **Patients (no previous RCT):**  Male: 8 Female: 18   **Research nurses:**  Male: 2 Female: 24 | Not reported | Questionnaire – Patient Understanding of Research | Not Reported |
| 66 | Jefford et al. 2011 (92) | Median: 58.4 Mean: 57.7 Range: 29–85 | Male: 68 (66.7%) Female: 34 (33.3%) | Primary: 2 (2.0%) Secondary up to year 9: 13 (12.7%) Secondary years 10, 11, or 12: 37 (36.3%) Trade qualifications: 17 (16.7%) Certificate/diploma: 10 (9.8%) University: Graduate degree: 16 (15.7%) University: Postgraduate degree: 6 (5.9%) No response: 1 (1.0%) | Quality of Informed Consent (QuIC) | objective and subjective understanding |
| 67 | Jeong et al. 2012 (93) | **Overall:** Mean: 52.6 (SD = 12.9) **Nurses:** Mean: 27.0 (SD = 4.9) | **Patients:**  Male: 34.9% Female: 64.2% No response: 0.9%   **Nurses:**  Male: 0.6% Female: 99.4% | **Patients:**  Middle school and below: 103 (24.1%) High school: 207 (48.5%) 3-year college: 116 (26.9%) No response: 2 (0.5%)  **Nurses:** 3-year college: 59 (37.3%) 4-year college: 86 (54.4%) Graduate school: 13 (8.2%) | A structured questionnaire developed by the research team | clinical related and adverse events related terms (Exposure of Perceived Understanding as: Don’t Know; Know; Vaguely; Know Well) |
| 69 | Joffe et al. 2001 (94) | Mean: 55 <45: 42 (20%) 45–64: 117 (57%) ≤65: 48 (23%) | Women: 114 (55%) | 53% college education | QuIC (Quality of Informed Consent) questionnaire | **13 independent domains of informed consent** 1. A statement that the study involves research 2. An explanation of the purposes of the research 3. The expected duration of the subject's participation 4. A description of the procedures to be followed 5. Identification of any procedures that are experimental 6. A description of any reasonably foreseeable risks or discomforts to the subject 7. A description of any benefits to the subject that may reasonably be expected from the research 8. A description of any benefits to others that may reasonably be expected from the research 9. A disclosure of appropriate alternative procedures or courses of treatment, if any, that might be advantageous to the subject 10. A statement describing the extent, if any, to which confidentiality of records identifying the subject will be maintained 11. For research involving more than minimal risk, an explanation as to whether any compensation and an explanation as to whether any medical treatments are available if injury occurs and, if so, what they consist of or where further information may be obtained 12. An explanation of whom to contact for answers to pertinent questions about the research and research subjects' rights and of whom to contact in the event of a research-related injury to the subject 13. A statement that participation is voluntary, refusal to participate will involve no penalty or loss of benefits to which the subject is otherwise entitled, and the subject may discontinue particpation at any time without penalty or loss of benefits to which the subject is otherwise entitled |
| 68 | Joffe et al. 2001 (18) | Mean: 55 | Female: 55% | college education: 53% | QuIC questionnaire | basic elements of informed consent specified in US federal regulations |
| 70 | Juan Salvadores et al. 2022 (95) | Mean: 61.46 (SD = 11) | Male: 175 (78.5%) Female: 48 (21.5%) | Incomplete primary education: 85 (38.1%) Primary education: 72 (32.3%) Medium education: 39 (17.5%) Higher education: 25 (11.2%) DK/NA/REF: 2 (0.9%) | Belmont Beta Questionnaire | 15 questions were related to patient comprehension, 12 for given information and 4 for patient willingness (voluntariness). |
| 71 | Juraskova et al. 2008 (96) | Mean: 60 | Female: 100% | post-graduate qualifications: 2; professional occupations: 15 | (1) Clinical trial knowledge sclae adapted form of a 7-item scale about trial methods and rationale for them ; (2) QuIC part B; (3) purpose-designed 13-item scale to assess understanding of the trial (IBIS-II DCIS trial understanding scale), (4) Attitude scale, (5) Decisional Conflict Scale (DCS), (6) Decision aid feedback measure | not stated |
| 72 | Karlawish et al. 2002 (97) | Patients: Mean: 72 (SD = 8.1) Range: 56–84 Controls: Mean: 77 (SD = 4.5) Range: 69–86 Caregivers: Mean: 64.9 (SD = 12.4) Range: 34–82 | Not reported | **Patients**: 15.3 ± 3.6 (8–20) years **Controls:** 15.9 ± 2.6 (10–20) years **Caregivers**: 15.8 ± 2.6 (10–18) years | MacArthur Competency Assessment Tool for Clinical Research (MacCAT-CR). (The interviewer adapted the MacCAT-CR questions) | "Understanding", "Appreciation", "Reasoning", "Choice" |
| 73 | Kashur et al. 2023 (98) | Median: 54 | Male: 15 (83.3%) | college education: 50% | Two survey questionnaires (not specifically named) | **Objective understanding** Purpose of consent Purpose of study Duration of study Nature of study intervention Number of study groups Understanding of alternative treatments Randomization Blindness Side effects Contacts in case of side effects Compensation in case of harm Voluntariness of withdraw Treatment options if refused to participate Benefits of participation Financial benefits of participation Confidentiality Whom to contact for any complaints |
| 74 | Kass et al. 2009 (99) | Not reported | Not reported | Not reported | structured questionnaire | beliefs about the purpose of the research, expected benefits and risks, and intended decision about enrollment |
| 75 | Kass et al. 2015 (100) | Mean: 51.5 Median: 52 | Female: 67.4% | Third grade or below: 2 (1.4%) Fourth to sixth grade: 5 (3.5%) Seventh to eighth grade: 27 (18.8%) High school: 110 (76.4%)​ | Consent Understanding Evaluation (CUE). | 25 questions related to study purpose, voluntariness, risks, and design; 3 additional questions were added for studies that were randomized. 8 attitudinal questions about research and consent; 8 questions about consent for this collaborating study; 11 demographic/background questions. |
| 76 | Kim & Kim 2015 (101) | Mean: 51.3 (SD = 13.9) | Female: 82 (54.7%) | Elementary: 19(12.6%); Middle: 28 (18.7%) High: 52 (34.7%) College: 51 (34.7%) | modified QuIC | clinical trial purpose, random assignment, procedure, terms of compliance, benefits and risks, costs and compensation, and voluntary participation and withdrawal |
| 78 | Knapp et al. 2009 (102) | Mean: 29 Range: 19–38 | Male: 100% | Higher education graduates: **first round:** 3/10 **later rounds**: 6/20 | 21-item questionnaire | 21 items covering the nature and purpose of the trial (2 questions); • the process and meaning of consent (7); • trial procedures (8); • safety and efficacy of the tested medicine (4). |
| 77 | Knapp et al. 2009 (103) | **Original sheet testing**: Mean: 34.3 Range: 23–44 Median: 36 **Revised sheet testing:** Mean: 33.5 Range: 23–41 Median: 34.5 | Female: 100% | higher education graduates: 9/20; unemployed or had occupations that did not involve regular use of written documents: 8/20 | 21-item questionnaire | the nature and purpose of the trial (2 questions); the nature and purpose of the trial (three questions); the process and meaning of consent (six); trial procedures (seven); safety and efficacy of the tested medicine (five). |
| 79 | Koh et al. 2012 (104) | Mean: 39.84 (SD = 17.42) Range: 20–75 | Men: 111 (91.74%) Women: 10 (8.26%) | Less than college: 30 (25%) college or post graduate: 90 (75%) | Modified version of Quality of Informed Consent (QuIC) questionnaire | Purpose of the research', ‘voluntariness of participation’, ‘duration of the research’, ‘study involves research’, ‘who tocontact’, ‘confidentiality’, ‘compensation’, ‘benefits to the subject’, ‘procedures’, ‘benefits to others’ and ‘risks or discomforts’. |
| 80 | Koonrungsesomboon et al. 2017 (105) | Mean: 50.5 (SD = 15.2) | Female: 61.2% | **SIDCER ICF group:** Level 1: 56.2%; Level 2: 41.5%; Level 3: 2.3%  **Conventional ICF group:** Level 1: 49.2%; Level 2: 46.1%; Level 3: 4.7% | post-test questionnaire; | General items; Rights of the subject; Scientific aspects; Ethical aspects |
| 81 | Kripalani et al. 2008 (106) | Median: 64 | Male: 45.3% Female: 54.7% | Mean: 10.9 years (SD = 3.2) | Teach back technique | Purpose of study, timing of follow-up, study groups, risks, benefits, records access, regulatory agencies' access, withdrawal procedures |
| 82 | Krosin et al. 2006 (107) | Not reported | **Village R:** Male: 92% Female: 8%  **Village U:** Male: 13% Female: 83% | **Village R:** not attended school and were illiterate: >90% **Village U:** primary education: 67%, literate to some degree: 70% | nine-item questionnaire | Voluntary participation Compensation Withdrawal criterion Withdrawal consequence Study versus treatment Study administration Randomization and placebo Side effects Lay scientific knowledge |
| 83 | Kruse et al. 2000 (108) | Mean: Control: 48 (SD = 18), leaflet 50 (SD = 20), brochure 46 (SD = 19), booklet 45 (SD = 19) | % male by arm: control 36 %, leaflet 40 %, brochure 41 %, booklet 42 % | **Control group:** Primary school 18%; Secondary school 54%; Higher education 28%  **Leaflet group:** Primary school 20%; Secondary school 46%; Higher education 34%  **Brochure group:** Primary school 23%; Secondary school 44%; Higher education 33%  **Booklet group:** Primary school 21%; Secondary school 56%; Higher education 23% |  |  |
| 84 | Länsimies-Antikainen et al. 2010 (109) | Mean: 66 (SD = 5) Range: 57–78 | Male: 586 (49%) Female: 609 (51%) | No professional training: 292 (24%) Vocational school or vocational course: 415 (35%) College-level training: 314 (26%) Academic degree: 173 (15% | Questionnaire developed by the authors | background information (14 questions); information, under- standing and competence (16 questions); and voluntariness and decision-making (14 questions). The questionnaire included 44 questions: multiple-choice questions, yes/no questions, short specifying open questions and a scale of 1–5 (Likert-scale). |
| 85 | Leach et al. 1999 (110) | Not reported | Not reported | **Rural:** Mothers: 20%; Fathers: 23% **Urban:** Mothers: 35%; Fathers: 55% | Semi-structured interview | 1. The families' experiences of the consent process. 2. Their knowledge of key aspects of the subject matter. 3. The motives for their decision. 4. People involved in the decision making. 5. Views on how the consent process should be conducted. |
| 86 | Lewis et al. 2015 (111) | Mean: 36.7 | Male: 31.4% Female: 68.6% | Did not complete high school: 29.4%; Completed high school/Received a GED: 31.4%; Additional professional/vocational training: 9.8%; Undergraduate education: 13.7%; Postgraduate education: 15.7% | Informed Consent Evaluation Survey | Can you get HIV from participating in the HIV vaccine trial? Yes, No, There is a small possibility  How can the HIV vaccine protect you against HIV infection? It contains a chemical that physically kills the virus It encourages your body to create immune cells that can kill HIV-infected cells It creates a physical protective coat over your body cells which prevents the virus from entering the cells and causing infecting It directly kills infected cells which are then excreted or recycled by the body I am not sure how the vaccine works  What is the primary ingredient in the HIV vaccine? Live HIV virus Dead HIV virus A weakened form of the HIV virus A protein component of the HIV virus. A non-organic anti-virus chemical I am not sure  What are the possible side effects from participating in the HIV vaccine trial? (Please check all that apply) A rash and/or swelling at the injection site Fever Nausea High Blood pressure Increased blood toxicity Death I am not sure what the possible side effects are |
| 87 | MacQueen et al. 2014 (112) | Mean: 26.7 (SD = 4.7) Median: 26 Range: 18–35 | Female: 100% | no school or primary school only: 53 (66.3%) higher than primary school education: 27 (33.8%) | Closed-ended comprehension assessment (IC-C) Open-ended comprehension assessment (IC-O) Self-perception comprehension assessment (IC-SP) | purpose of the research, study procedure, possible risks, possible benefits, confidentiality, Contacts for questions about research and rights; Voluntariness |
| 88 | Mansour et al. 2015 (113) | < 40: 27 (26.2%) 40–55: 49 (47.6%) 55: 25 (24.3%) Missing data: 2 (2.0%) | Male: 54 (52.4%) Female: 49 (47.6%) | Illiterate: 17 (16.5%) High school or less: 52 (50.5%) Higher education: 33 (32.0%) Missing data: 1 (1.0%) | Questionnaire to assess participant perspective | views regarding the informed consent process; evidence of therapeutic misconceptions; and motivation for participation |
| 89 | Mboizi et al. 2017 (114) | 18–24: 39.0% 25–29: 27.6% 30+: 33.4% | Female: 100% | 0 years: 17.2%; 1-5 years: 23.9%; 6-10 years: 37.1%; 11-14 years: 19.9%; 15+ years: 1.9% | Assessment of Consent recall Questionnaire (ACQ) Additional Questionnaire (AQ) | Not explicitly stated |
| 90 | Meneguin et al. 2014 (115) | Group I: Mean: 54.2 (SD = 8.0) Group II: Mean: 54.6 (SD = 8.4) | Male: 10 (52.6%) Female: 9 (47.4%) | Most participants were illiterate or had only incomplete primary level of education | Focus group discussion guide with 8 questions | experiences, values, beliefs and attitudes about specific issues |
| 91 | Mexas et al. 2014 (116) | Mean: 36 (SD = 15) Median: 32 (IQR: 24–48) | Men: 40 (65%) Women: 21 (35%) | 0–8 years: 31 (51%) 9–12 years: 18 (29%) 12 years and more: 12 (20%) | informed consent assessment instrument (ICAI) | participant’s awareness of enrollment in a clinical trial (question 1), the use of two different treatments (question 2), random selection of the treatment arm (question 3), the diagnostic procedures and tests at baseline (questions 4 and 5), the autonomy of the participant (question 6), the clarity of the explanation of risks and benefits of study participation (question 7), the risks of the study drugs on contraception and exclusion of pregnant women from the study (questions 8 and 9), and nonpayment for participating in the study (question 10) |
| 92 | Miller et al. 1994 (117) | Mean: 30 (SD = 12.4) Range: 19–71 | Male: 68 (40.5%) Female: 100 (59.5%) | Mean: 15 years (SD 2.0) Range: 9–23 years | Patient Interview Questions | (1) understanding, (2) the decision making process, including the adolescent’s role, impact of faith on the decision, and perceived pressure, (3) expectations regarding the effect of trial participation on quality and length of life, and (4) reasons for agreeing to or declining the Phase I study. |
| 93 | Miller et al. 1996 (118) | Mean: 36 (SD = 12.8) Range: 18–78 | Men: 103 (32%) Women: 172 (68%) | Range: 10-24 years (mean ± SD 14.4 ± 2.3 yrs), which translates to an average of 2 years of postsecondary education.  High school: 26%; college degree: 28% | 14 item questionnaire | Seven questions (1, 2, 7, 9, 10, 11, 13) were designed to assess whether the subjects recalled participating in the study, receiving an informed consent document, the names of study medications, side effects, and so forth. If a participant could not accurately recall the names of the study medications or side effects, their ability to recognize the information was assessed using a list containing both correct and incorrect items (questions 8 and 12). The remaining questions (3, 4, 5, 6, and 14) were designed to assess their perceptions regarding their understanding of the study, accuracy of the consent form, and how well they felt their human rights were protected; three of these questions were answered using a seven-point Likert scale. |
| 94 | Miller et al. 2013 (119) | Mean: 17.8 (SD = 2.35) Range: 14–21 | Male: 15 (75%) | Not reported | Deaconess Informed Consent Comprehension Test (DICCT). | The eight basic elements of informed consent as mandated by US regulations |
| 95 | Mills et al. 2003 (120) | Range: 50–69 | Male: 100% | a range of educational backgrounds | patient interview guide | understanding of the purpose of the ProtecT study, the treatments involved, their recall and understanding of the study design (including randomization, the involvement of chance, the existence of equipoise/uncertainty, and beliefs about the method of treatment allocation), the acceptability of the treatment decision reached, and the factors involved in their decision to accept or reject randomization and/or treatment allocation |
| 96 | Minnies et al. 2008 (121) | Median: 26 Range: 16–44 | Female: 100% | Grade 6 or less: 23.4%; Grade 7 to 11: 42.8%; Grade 12 or higher: 33.9% | Quality of consent evaluation questionnaire | key elements of informed consent (voluntary participation, confidentiality, the main risks and benefits, etc.). The recall (success in selecting the correct answers) and understanding (correctness of interpretation of statements presented) were measured. |
| 97 | Montgomery et al. 1998 (122) | **Ranges in 6 trials:** 4–12, 28–65, 25–61, 56–76, 25–59, 26–75 | Male/Female (in 6 trials): 24/25, 0/50, 0/60, 8/1, 3/15, 10/8 | Not reported | Postal Questionnaire | understanding, voluntariness, and pressure to participate |
| 98 | Moodley et al. 2005 (123) | Mean: 68 Range: 60–80 | Women: 69% | Median: grade 8 (range: 0-12 grade) | Semi-structured questionnaire | purpose of the study; awareness that the study was not part of routine treatment; voluntary nature of participation and freedom to withdraw; randomisation; placebos; and remuneration. |
| 99 | Nguyen et al. 2023 (124) | **Trial participants:** Median: 50 Range: 24–67 Up to 30: 3 (12%) 31–50: 8 (32%) 51–60: 7 (28%) Above 60: 7 (28%)  **Study physicians:** Up to 30: 2 (28.6%) 31–45: 5 (71.4%) | **Trial participants:** Male: 9 (36%) Female: 16 (64%)   **Study physicians:**  Male: 3 (42.9%) Female: 4 (57.1%) | No schooling: 0 (0%) Grade 1—5: 8 (36.4%) Grade 6—9: 7 (31.8%) Grade 10—12: 5 (22.7%) University or above: 2 (9.1%) | in-depth interview guide (for healthcare workers) in-depth interview guide (for patients) Observation guides | to explore the experiences and perceptions regarding consent seeking/giving, issues of comprehension of the information provided about clinical trials during the consent process, and reasons for joining or refusing to join a clinical trial.  The observation guide included sections for descriptions related to duration, space and atmosphere, content of discussion and interactions between the physicians and the trial participants. |
| 100 | Norris et al. 1990 (125) | Not reported | Not reported | Not reported | Knowledge test/ Patient consent quiz | Not Reported |
| 101 | O’Sullivan et al. 2022 (126) | Not reported | Not reported | Not reported | modified version of the ‘‘quality of informed consent measure’’ (QuIC) | Nature of research, purpose, duration, potential risks and discomfortS, benefits, confidentiality, procedure in the event of injury, study contacts, voluntariness, procedures to be followed, experimental nature of study, Alternative to participation |
| 102 | Ormond et al. 2009 (127) | Mean: 50.5 | Female: 132 (66.0%) | college degree: 68.5% | 10-question test | purpose of study; who can take part; risks and benefits; right to withdraw; benefits of taking part; key contact for info about study; confidentiality; purpose of samples taken. |
| 103 | Ossemane et al. 2018 (128) | Median: 27 (IQR: 22–32) | Male: 5% Female: 95% | No formal education: 34 (25%) Primary: 58 (42%) Secondary: 39 (28%) Pre-University: 6 (4%) University: 1 (1%) | Modified Deaconess Informed Consent Comprehension Test (DICCT) | Comprehension and recall of informed consent information |
| 104 | Paris et al. 2015 (129) | **Original ICD group:** Mean: 46.8 (SD = 19.4) **Modified ICD group:** Mean: 46.7 (SD = 18.2) | **Original ICD group:** Male: 142 (59.4%) **Modified ICD group:** Male: 97 (40.6%) | **Original:** School leaving certificate: 23.6% High school graduation: 18.8% Undergraduate degree: 38.8% Postgraduate degree: 18.8%  **Modified:** School leaving certificate: 17.4% High school graduation: 26.0% Undergraduate degree: 40.8% Postgraduate degree: 15.8% | American Quality of Informed Consent questionnaire (QuIC questionnaire) | Objective comprehension, subjective comprehension |
| 105 | Penn et al. 2010 (130) | Range: 18–22 | Not reported | University students | student comprehension tests and semi-structured interview | Not Reported |
| 106 | Ponzio et al. 2018 (131) | Mean: 52.9 (SD = 11.7) | Not explicitly stated, but mentioned that "The groups were compatible for gender" | Not reported | 21-item ad hoc user testing questionnaire | the nature and purpose of the trial, the process and meaning of the informed consent, trial procedures and safety, and efficacy of the treatment |
| 107 | Pope et al. 2003 (132) | Median: 63 Range: 22–84 | Male: 84 (45%) Female: 106 (55%) | Elementary: 35 (18%) High school: 86 (45%) Higher education: 69 (36%) | Questionnaire developed for this study | recall and understanding of information and trial concepts, subjects’ decisions to participate, and perceptions/opinions of the study |
| 108 | Ranjan et al. 2019 (133) | 18–28: 240 (60.0%) 29–38: 146 (36.5%) 38+: 14 (3.5%) | Male: 356 (89.0%) Female: 44 (11.0%) | Illiterate: 25 (6.2%) Primary school or literate: 25 (6.2%) Middle school completion: 174 (43.5%) High school certificate: 104 (26.0%) Intermediate: 66 (16.5%) Graduation and more: 6 (1.5%) | not specified | Awareness of informed consent process and participant rights Attitude towards undergoing, receiving, retaining and referring to informed consent forms Actual practice of informed consent process |
| 109 | Ravina et al. 2010 (134) | Mean: 61.4 (SD = 10.0) | Male: 65.8% Female: 34.2% | Mean: 15.3 years (SD 3.3) | 30 item multiple-choice questionnaire | purpose of study, random allocation of treatment, experimental nature of drugs, voluntary participation, expected effect on their PD, and placebo concept. |
| 110 | Rikkert et al. 1997 (135) | Mean: 80.1 Range: 70–92 | Men: 17 Women: 36 | completed primary school: 29 | Comprehension assessment adapted from Miller's "two-part consent form" | benefits, departure from ordinary medical practice, risks, inconveniences and tasks, purposes, and the subjects' rights |
| 111 | Rose et al. 2013 (136) | **London site**: Mean: 45.92 (SD = 11.95) **Mannheim site:** Mean: 42.47 (SD = 12.35) **Aarhus site:** Mean: 39.95 (SD = 12.11) **Poznan site:** Mean: 41.89 (SD = 12.52) | Males/Females by site: **London:** 8/10 **Mannheim:** 8/11 **Aarhus**: 4/15 **Poznan:** 6/14 | Not reported | Semi-structured interview guide | purpose of the trial; Consent; DNA/blood sampling; Lack of choice of medication; Completion of trial |
| 112 | Roth et al. 2021 (137) | Mean: 66.8 (SE = 8.1) Median: 67.0 (IQR: 10.4) | Male: 57.3% | 0-11 years: 19 (9.2%) High-school graduate: 67 (32.5%) Some college or technical school: 68 (33.0%) College graduate (4-year college or bachelor's degree): 30 (14.5%) Graduate school or advanced degree: 22 (10.7%) | 38-item telephone survey | to Inform Treatment Eligibility in Lung-MAP, to Confirm Diagnosis of Lung Cancer, to Inform Personal Risk of Other Diseases, to Inform Family Members’ Risk of Developing Cancer |
| 113 | Ruiz De Hoyos et al. 2020 (138) | Mean: 59.2 (SD = 17.3) | Male: 16 (50%) | Not reported | Newly developed questionnaire including adapted Quality of Informed Consent (QuIC) questionnaire | Part 1: 1. Motivations to participate 2. Appraisal of the briefing process 3. Appraisal of the decision-making process 4. Expectations from the study Part 2: 1. Experimental nature of the study 2. Process 3. Benefits 4. Risks and inconveniences 5. Procedures used  6. Alternatives 7. Confidentiality 8. Voluntariness 9. Aspects related to therapeutic misconception |
| 114 | Russel et al. 2005 (139) | **Aboriginal participants**: >16: 19/20, <16: 1/20 **non-Aboriginal participants**: >16: 20/20 | Female: 100% | **Aboriginal group:** High school only: 1 (5%) Adult training: 19 (95%)  **Non-Aboriginal group:** High school only: 4 (20%) Adult training: 16 (80%) | Questionnaire (not named) | Understanding of diseases prevented by vaccine, licensed vs unlicensed vaccine concept, potential risks, voluntary nature of participation |
| 115 | Sanchini et al. 2014 (140) | Mean: 66.4 (SD = 8.8); Range: 42–83 | Male: 49 (64%) Female: 28 (36%) | <High school: 37 (49%) High school: 25 (33%) University: 14 (18%) | Ad hoc questionnaire specifically devised for this survey | Awareness of participating in a clinical trial, understanding of study procedures, risks, benefits, randomization, etc. |
| 116 | Sand et al. 2008 (141) | Median: 69; Range: 44–84 | Men: 12 Women: 9 | Primary and/or lower secondary school: 10 Between 1 and 3 years of upper secondary education: 9 More than 4 years of higher education: 2 | Semi-structured interview guide designed for this study | Not Reported |
| 117 | Sarkar et al. 2010 (142) | Mean: 29.3 (SD = 7.3) | Female: 113 (95.8%) | No formal education: 21 (17.8%) Did not finish high school (year 10): 101 (85.6%) | Structured questionnaire | Understanding of study purpose, randomization, risks/benefits, voluntary participation, freedom to withdraw |
| 118 | Schats et al. 2003 (143) | Not reported | Not reported | Not reported | Standardized questionnaire (not named) | explanation about the disease; the name, effect, and side effects of the drug; the route of administration of the drugs; the placebo controlled and double blind design of the trial; the voluntary nature of participation; privacy regulation with regard to the case record forms; the duration of the trial; and the right to withdraw at any moment. |
| 119 | Schmanski et al. 2021 (144) | 19–39: 235 40–59: 292 60+: 193 | Male: 249 Female: 497 | High school or high school equivalent: 93; Associates degree: 90; Bachelors degree: 271; Graduate or professional degree: 292 | Custom survey developed by the researchers | purpose of the Biobank, voluntary nature of the study, methods of blood sample collection, who will perform this research, and the small risk of loss of confidentiality |
| 120 | Schumacher et al. 2017 (145) | < 60: 25 (46%) ≥ 60: 29 (54%) | Men: 30 (56%) Female: 24 (44%) | Less than high school diploma 5 (9%) High school diploma: 22 (41%) Associate degree: 7 (13%) Bachelors degree: 14 (26%) Masters degree: 6 (11%) | 14-question survey (modified version of the QuIC survey i.e. QuIC-A) | 1) explanation of the purpose and procedures of the research, 2) description of foreseeable risks or discomforts, 3) benefits to self and others, 4) disclosure of alternative treatments, 5) confidentiality of records, 6) explanation of compensation or treatment in the event of research-related injury, 7) contact information in case of research-related questions or injury, and 8) a statement reinforcing voluntary participation |
| 121 | Searight et al. 1996 (146) | Mean: 40.5 | Men: 3 Women: 11 | Mean: 14.4 years of formal education | Semi-structured qualitative interview | Motivation for participation, protection of human rights, understanding of placebos and randomization, perceptions of consent forms, distinction between personal care and research |
| 122 | Sengupta et al. 2011 (147) | **Intervention group:** Mean: 39 (SD = 8) **Delayed intervention group:** Mean: 36 (SD = 10) | **Intervention group:** Male: 10 (83%) Female: 2 (17%)   **Delayed intervention group:** Male: 9 (100%) | **Intervention group:** 11th grade and under: 2 (17%) High school/G.E.D: 2 (17%) Some college or training: 5 (42%) College degree: 1 (8%) Some graduate school or graduate degree: 2 (17%) **Delayed intervention group:** 11th grade and under: 1 (11%) High school/G.E.D: 2 (22%) Some college or training: 2 (22%) College degree: 1 (11%) Some graduate school or graduate degree: 3 (33%) | Adapted version of the Quality of Informed Consent (QuIC) measure | **8 basic elements of informed consent:**  Explanation of the purposes, description of the research, along with subjects' expected participation Description of any reasonably foreseeable risks or discomforts to subjects Description of any benefits to subjects Disclosure of appropriate alternative procedures or courses of treatment Information related to the confidentiality of data For research involving more than minimal risk, an explanation of any compensation with respect to injury Explanation of whom to contact for answers to pertinent questions about the research and research subjects' rights A statement that participation is voluntary |
| 123 | Shafiq et al. 2011 (148) | Not reported | Not reported | Not reported | 24-item questionnaire | Background of the study, Study design, Participants' rights, Miscellaneous aspect |
| 124 | Shamy et al. 2019 (149) | Range: 40–80 (82% of respondents) | Male: 58% | Not reported | 12-question survey | Understanding of study purpose/design, opinions on appropriateness of deferred consent |
| 125 | Shelton et al. 2015 (150) | **Experimental group:** Mean: 49.4 (SD = 15.35) **Control group:** Mean: 45 (SD = 15.53) | **Experimental group:** Male: 31% Female: 69%   **Control group**:  Male: 36% Female: 64% | **Experimental group:** Less than high school: 3%; High school graduate: 23%; Some college: 35%; College graduate: 34%; Postgraduate college: 5%  **Control group:** Less than high school: 1%; High school graduate: 28%; Some college: 35%; College graduate: 26%; Postgraduate college: 10% | 13-item posttest instrument | 1. Intended benefit for future patients 2. Purpose of surrogate consenting 3. Study withdrawal 4. Purpose and length of the study 5. Overall research risks 6. Purpose of the institutional review board 7. Need for and purpose of researcher’s contact information 8. Sufficient information to make an informed decision 9. The voluntary nature of research 10. Substituted judgment 11. Confidentiality of information 12. Compensation for harm 13. Alternative treatment |
| 126 | Shiono et al. 2014 (151) | Mean: 43.8 (SD = 3.1) | Female: 100% | Junior high school: 3 (0.8%) High school: 96 (25.5%) Junior college: 154 (41.0%) University, graduate school: 105 (27.9%) No reply: 18 (4.8%) | Japanese version of the Quality of Informed Consent (QuIC) scale | Objective understanding and subjective understanding |
| 127 | Siao et al. 2014 (152) | Mean: 55.5 (SD = 0.6) | Not reported | None: 4 (1.65%) Elementary: 72 (26.7%) High school: 114 (40.9%) College: 80 (27.3%) Graduate: 23 (7.8%) | Multilingual survey developed by the authors | The survey assessed whether the following aspects were explained: reasons for procedure, alternatives, risks, what provider will do, benefits, what to expect after, right to refuse. It also included questions on adequacy of information, understanding, and satisfaction. |
| 128 | Smith et al. 2016 (153) | Age groups: <35: 35.2% 35–44: 57.1% | Female: 100% | Did not finish high school: 9 (4.2%) High school certificate: 22 (10.4%) Vocational training: 42 (20%) University: 121 (57.6%) Mising: 16 (7.6%) | Anonymous self-completion questionnaire (no specific name given) | Understanding of study information Recall of study procedures Comprehension of research terminology Satisfaction with information provided |
| 129 | Spellecy et al. 2011 (154) | **ETRIC group:** Median: 61; Range: 21–76 **Standard consent group**: Median: 61; Range: 27–73 | **ETRIC group**:  Male: 57 (57%) Female: 43 (43%)   **Standard consent group**:  Male: 53 (54%) Female: 45 (46%) | **ETRIC group:** High school or less: 35 (35%) More than high school but less than graduate degree: 27 (27%) Graduate degree or more: 11 (11%) Other: 6 (6%) Declined/missing: 21 (21%)  **Standard consent group:** High school or less: 20 (20%) More than high school but less than graduate degree: 29 (30%) Graduate degree or more: 15 (15%) Other: 5 (5%) Declined/missing: 29 (30%) | Quality of Informed Consent (QuIC) Modified Deaconess Informed Consent Comprehension Test State Trait Anxiety Inventory (STAI) Study-specific satisfaction survey Study-specific information location instrument | **interview domains** Willingness touseandacceptability to ETRIC form, Perceived value of ETRIC form, Perceived barriers to implementation of ETRIC form, Previous experience with alternative consent forms, Educational resourcesfor implementing ETRIC |
| 130 | Sudore et al. 2006 (155) | Mean: 61 (SD = 8.6) | Female: 53% | Less than high school 32% High school 19% College graduate 49% | teach-to-goal consent process (consent form and comprehension statements) | study procedures, risk, and confidentiality |
| 131 | Tadros et al. 2019 (156) | Mean: 33; Range: 18–84 | Female: 40%-61% | Not reported | Questionnaire developed by the researchers (no specific name given) | Not explicitly stated, but dimensions covered include study purpose, drug information, administration details, prior exposure, side effects, and payment |
| 132 | Tait et al. 2003 (157) | Mean: 37.3 (SD = 7.3) | Not reported | ≤ High school graduate: 120 (24.4%) Some college: 129 (26.2%) ≥ College graduate: 243 (49.4%) | Questionnaire and semi structured interview | Study purpose , Protocol, Risks, Direct benefits, Indirect benefits, Freedom to withdraw, Alternative treatments or procedures, Voluntariness, Duration of participation, Contact , Confidentiality |
| 133 | Taiwo et al. 2009 (158) | Mean: 46.1 (SD = 16.3); Range: 21–80 | Male: 79 (69.9%) Female: 34 (30.1%) | None: 37; Primary: 31; Secondary: 33; Tertiary: 12 | Quantitative survey (About 80% of the survey questions were taken from QuIC and DICCT) and Qualitative semi-structured interview | knowledge of being in a research study, understanding of the purpose of the parent study, risks, benefits, confidentiality, voluntariness, and whom to contact in case of any questions. |
| 134 | Taylor et al. 2021 (159) | Mean: 54 Median: 56 | Male: 169 (62%) Female: 102 (37.4%) Other: 2 (0.7%) | Less than high school: 14% (n = 38) 12th grade/GED: 25.3% (n = 69) Associate/technical degree/some college: 22% (n = 60) College degree: 19.8% (n = 54) Some graduate school: 1.8% (n = 5) Graduate school degree: 17.2% (n = 47) | Consent Understanding Evaluation—Refined (CUE-R) | too long, attach as document |
| 135 | Tindall et al. 1994 (160) | Median: 39; Range: 27–54 | Male: 100% | high school education: 100%; university studies: 52 (49%) | Eight-item true/false/don't know questionnaire about ddI | Not reported |
| 136 | Van Den Bergh et al. 2009 (161) | Participants: Mean: 57.7 (SD = 5.6); Median: 56.6 Nonparticipants: Mean: 59.3 (SD = 6.2); Median: 58.6 | **Participants:** Male: 48.5% Female: 51.5%   **Nonparticipants:**  Male: 33.0% Female: 67.0% | **Participants:** Primary education: 8.6% Lower vocational or lower secondary general education: 41.3% Intermediate vocational or higher secondary general education: 24.5% Higher vocational education or university: 25.6% **Nonparticipants:** Primary education: 15.5% Lower vocational or lower secondary general education: 38.1% Intermediate vocational or higher secondary general education: 22.7% Higher vocational education or university: 23.7% | Informed Decision-making (IDM) questionnaire | Knowledge about lung cancer, lung cancer screening, and trial characteristics Attitudes towards lung cancer screening Cognitive and affective risk perception Reasons for participation/nonparticipation Informed decision-making |
| 137 | Van Stuijvenberg et al. 1998 (162) | Median age in (25th–75th percentiles): **Mothers:** 32.6 (29.0–37.0) **Fathers:** 35.6 (31.6–39.5) | Male: 155 Female: 181 | **Mother (%):** Unknown: 2 (1%) Elementary school: 23 (13%) Lower general secondary education: 21 (12%) Vocational training (lower level): 31 (17%) Higher general secondary education/pre-university education: 23 (13%) Vocational training (intermediate level): 46 (25%) Vocational training (higher level): 29 (16%) University education: 6 (3%)  **Father (%):** Unknown: 4 (3%) Elementary school: 13 (8%) Lower general secondary education: 13 (8%) Vocational training (lower level): 34 (22%) Higher general secondary education/pre-university education: 17 (11%) Vocational training (intermediate level): 31 (20%) Vocational training (higher level): 34 (22%) University education: 10 (6%) | Questionnaire | **a. Awareness of six major trial characteristics**  -Aim of the study -Reason for signing the informed consent form -Possible negative side effects -50% chance of being assigned a placebo -Random allocation procedure -Possibility of withdrawing  **b. Major reasons for approval versus feeling obliged to participate** - Contribution to clinical science -Benefit for their own child -Benefit for other children in future  -Benefit for the parent -Give something in return for the care of their child -The doctor asked -No major reason  **c. Perception of the IC procedure** -Evaluation of invitation to participate -Sufficient time to decide -Sufficient explanation  **d. Advantage sand disadvantage of study  e. Willingness and reasons for participation in a similar future study** -Willing -not willing -no reason |
| 138 | Verheggen et al. 1996 (163) | Mean: 57.8 (SD = 13.8) | Male: 61.6% | Not reported | Custom questionnaire developed for this study | Information disclosure, written information, time to reconsider, risks/benefits/barriers perceptions |
| 139 | Vickers et al. 2021 (164) | Not reported | Male: 100% | Not reported | Quality of Informed Consent (QuIC) questionnaire | Knowledge (Part A) and subjective understanding (Part B) of consent process |
| 140 | Wade et al. 2009 (165) | 50–59: 6 60–69: 17 | Male: 100% | Not reported | audio-recordings of information appointments. | diagnosis, advantages and disadvantages of treatments, determining treatment outside the trial, the need for an RCT, the purpose of randomisation, the right to refuse participation or take time to consider, and inviting a decision as to how to proceed |
| 141 | Wada et al. 2017 (166) | Not reported | Not reported | Not reported | Participatory and Informed Consent (PIC) measure | .Consultation purpose  Relevant history, diagnosis and/or management to date  Current management options (independent of study)  Clinical Equipoise regarding trial treatments  Research study purpose or question  Trial arm 1 processes, disadvantages/risks, advantages/benefits  Trial arm 2 processes, disadvantages/risks, advantages/benefits  (3 arm trial only) Trial arm 3 processes, disadvantages/risks, advantages/benefits  Reason for trial or trial purpose  Randomisation  ◦ Reason for randomisation  ◦ Process of randomisation  Detail on trial treatment options  ◦ Processes, potential risks and benefit  Detail on trial procedures  ◦ Potential risks/costs/burden & benefits of taking part,  ◦ Options to refuse or withdraw,  ◦ Options for further support in decision making about participation,  ◦ Benefits to professional or organisation of P taking part.  ◦ Confidentiality of data  ◦ Explanation re compensation arrangements |
| 142 | Weston et al. 1997 (167) | **Video group:** Mean: 31.4; Range: 21.8–39.5 **Control group**: Mean: 31.8; Range: 18.0–37.3 | Women: 100% | **video gr:**  high school or lower: 4.8%; college or higher: 95.2% **control gr:**  high school or lower: 8.3%; college or higher: 91.7% | Questionnaire developed for the study (no specific name given) | (1) women’s willingness to participate: (2) their views that the Term PROM Study was worthwhile: and, (3) their ability to understand and retain information about: (a) prelabour rupture of the membranes at term; (b) the risks and benefits associated with the options for their care: and (c) various aspects of the study protocol. |
| 143 | Wirshing et al. 1998 (168) | Mean: 46.5 (SD = 8.5) | Male: 94% Female: 6% | Mean: 13.0 years (SD=1.8) | Informed Consent Survey | Understanding of study details, goals, risks, benefits, and patient rights |
| 144 | Woodward et al. 1979 (169) | Mean: 24; Range: 19–37 | Not reported | Mean: 13 years (range 6-17) | standard examination | general questions related to the subject of cholera (as opposed to research participation) |
| 145 | Yanics et al. 1996 (170) | Mean: 35.68 (SD = 12.70); Range: 18–78 | Male: 81 (45%) Female: 99 (55%) | Mean: 14.43 (SD =2.30), Range (10- 22) years | a. Deaconess Informed Consent Comprehension Test; b. Self-Rated Understanding scale | 1. purpose of study, 2. begin medication, 3. frequency of medication, 4. risk and side effects, 5. Benefits, 6. Alternative treatments, 7. contact about rights, 8. contact about injury/illness, 9. compensation available, 10. who pays compensatiob, 11. participation refusal, 12. withdraw any time, 13. consequences of withdrawal, 14. confidentiality |
| 146 | Young-Afat 2021 (171) | Not reported | Not reported | Not reported | questionnaire | Not Reported |
| 147 | Yuval et al. 2000 (172) | Not reported | Not reported | Not reported | the patient questionnaire | (1) explanation of the study and patient comprehension at the time of consent and randomization (questions 1-8), (2) feelings and reactions of the patient, family, and family practitioner during the study (questions 9-17), and (3) response and emotions of the patient after completion of the study (questions 18-22). |
| 148 | Zhang et al. 2017 (173) | **Video group:** 50–70: 29 (73%) 71–90: 11 (27%)  **Control Group:** 50–70: 27 (67%) 71–90: 13 (33%) | **Video group:**  Male: 21 (52%) Female: 19 (48%)   **Control Group:**  Male: 18 (45%) Female: 22 (55%) | **Video group** Illiterate: 5 (13%)  Primary education: 27 (67%) Secondary or higher education: 8 (20%)  **Control Group** Illiterate: 4 (10%) Primary education: 26 (65%) Secondary or higher education: 10 (25%) | 10-item questionnaire | surgical procedure and its risks, benefits, and reasonable alternatives aswell as the uncertainties endemic to each alternative |
