# Supplementary material for: Measuring and evaluating participant understanding of consent processes in clinical trials: a systematic review
Source: Trials. 2026 Mar 4;27:192. doi: 10.1186/s13063-026-09582-x (PMC12964858; doi:10.1186/s13063-026-09582-x)
Supplement: Supplementary file 3 — Supplementary Material 3. [file 13063_2026_9582_MOESM3_ESM.docx]

**Supplementary table 3**

| **S.No.** | **Tool name** | **Studies (reference)** | **Validity of measure/tool** | **Reliability of measure/tool** | **Mode of administration** | **Time required to complete** |
| --- | --- | --- | --- | --- | --- | --- |
| 1 | **Quality of Informed Consent (QuIC) Questionnaire** | Addissie et al. 2016 | ✘ | ✘ | questionnaire based interviews | Estimated 7.2 minutes to complete. |
|  |  | Barrett 2005 | ✘ | ✘ | self |  |
|  |  | Bergenmar et al. 2011 | ✘ | ✘ | self |  |
|  |  | Corneli et al. 2012 | ✘ | ✘ | nurse |  |
|  |  | Gad et al. 2022 | ✘ | ✘ | self |  |
|  |  | Gillespie 2017 | ✘ | ✘ | self |  |
|  |  | Golembiewsky et al. 2021 | ✘ | ✘ | tablet-based, self-guided |  |
|  |  | Gota et al. 2018 | ✘ | ✘ | trial coordinator |  |
|  |  | Hoffner et al. 2012 | ✘ | ✘ | phone call |  |
|  |  | Jefford et al. 2011 | ✘ | ✘ | self |  |
|  |  | Joffe et al. 2001 | ✔ | ✔ | self |  |
|  |  | Joffe et al. 2001 | ✘ | ✘ | self |  |
|  |  | Juraskova et al. 2008 | ✘ | ✘ | questionnaire based phone interviews |  |
|  |  | Paris et al. 2015 | ✘ | ✘ | self |  |
|  |  | Spellecy et al. 2011 | ✘ | ✘ | self |  |
|  |  | Vickers et al. 2021 | ✘ | ✘ | self |  |
| 2 | **Modified Quality of Informed Consent (QuIC) questionnaire** | Alexa-Stratulat et al. 2018 | ✘ | ✘ | self | ✘ |
|  |  | Atal et al. 2018 | ✘ | ✘ | self |  |
|  |  | Bergenmar et al. 2008 | ✘ | ✘ | self |  |
|  |  | Brandberg et al. 2016 | ✘ | ✘ | self |  |
|  |  | Ditae et al. 2018 | ✘ | ✘ | Open-Data Collection Kit forms on smartphones |  |
|  |  | Ford et al. 2008 | ✘ | ✘ | cognitive interview |  |
|  |  | Kim & Kim 2015 | ✘ | ✔ | self |  |
|  |  | Koh et al. 2012 | ✘ | ✘ | self |  |
|  |  | Taiwo et al. 2009 | ✘ | ✘ | interviewer |  |
|  |  | Ormond et al. 2009 | ✘ | ✘ | telepphone interview | 20 minutes |
|  |  | Ruiz De Hoyos et al. 2020 | ✔ | ✘ | self | 16.6 minutes (range: 14-20) |
|  |  | Schumacher et al. 2017 | ✘ | ✘ | self | without a time limitation |
|  |  | Sengupta et al. 2011 | ✘ | ✘ | In-person at baseline, telephone for follow-ups. | Baseline: 15-20 minutes Follow-ups: 10-15 minutes |
|  |  | Shiono et al. 2014 | ✘ | ✔ | self | within 2 weeks |
| 3 | **Deaconess Informed Consent Comprehension Test (DICCT).** | Miller et al. 1996 | ✔ | ✔ | study investigator | 7 mins |
|  |  | Boyd et al. 2021 | ✘ | ✘ | interview via telephone | ✘ |
|  |  | Yanics et al. 1996 | ✘ | ✘ | study investigator |  |
| 4 | **Modified Deaconess Informed Consent Comprehension Test (DICCT)** | osullivan et al. 2022 | ✘ | ✔ | Face-to-face in clinic or by phone | ✘ |
|  |  | Rikkert et al. 1997 | ✘ | ✘ | researcher |  |
|  |  | Spellecy et al. 2011 | ✘ | ✘ | self |  |
|  |  | Tait et al. 2003 | ✘ | ✔ | questionaire based interview |  |
|  |  | Taiwo et al. 2009 | ✘ | ✘ | administered by an interviewer |  |
| 5 | **Modular Informed Consent Comprehension Assessment (MICCA) & Brief Investigator Questionnaire (BIQ)** | Addissie et al. 2016 | ✔ | ✔ | questionnaire based interviews | ✘ |
| 6 | **Digitised Informed Consent Comprehension Questionnaire (DICCQ)** | Afolabi et al. 2014 | ✔ | ✔ | interviewers via laptop (in person) | test = 22.4±7.4 min retest = 18.5±5.4 min. |
|  |  | Afolabi et al. 2015 | ✘ | ✘ | interviewers via laptop (in person) | 32 minutes |
| 7 | **“teach-to-goal” or 'teach back technique'** | Ahalt et al. 2017 | ✘ | ✘ | by reading the consent form aloud to participants and asking them to describe the research procedures or answer questions about the study | ✘ |
|  |  | Kripalani et al. 2008 | ✘ | ✘ | Verbal administration by interviewer | about five minutes |
|  |  | Sudore et al. 2006 | ✘ | ✘ | participants were read comprehension statements and had to respond correctly to all statements | Participants were read the statements up to 3 times (specific time period not given) |
| 8 | **Informed Consent comprehension questionnaire** | Bhansali et al. 2009 | ✘ | ✘ | study coordinator | ✘ |
|  |  | Shafiq et al. 2011 | ✘ | ✘ | administered by trained personnel in local language |  |
| 9 | **21-item questionnaire** | Knapp et al. 2009 | ✘ | ✘ | questionnaire based interview | Mean 20.1 mins (range 15.7 - 26.0 mins, median 19.5 mins) |
|  |  | Knapp et al. 2009 | ✘ | ✘ |  | mean of 25 minutes (range 12-47 mins) |
|  |  | Ponzio et al. 2018 | ✘ | ✘ | self | ✘ |
| 10 | **Informed Consent Questionnaire (ICQ), reduced to ICQ-4** | Guarino et al. 2006 | ✔ | ✔ | self | ✘ |
|  |  | Guarino et al. 2006 | ✘ | ✘ |  |  |
| 11 | **University of California, San Diego Brief Assessment of Capacity to Consent Questionnaire (UBACC),** | Campbell et al. 2017 | ✔ | ✔ | with assistance from the nurse recruiters | 15 to 30 minutes. |
| 12 | **Consent quiz (CQ) instrument** | Allen at al. 2017 | ✔ | ✘ | self | ✘ |
| 13 | **English Questionnaire (by J. Collins)** | Collins et al. 2023 | ✘ | ✘ | self | within a few minutes |
| 14 | **Patient Survey** | Abd-Elsayed et al. 2012 | ✘ | ✘ | self | ✘ |
| 15 | **Anonymous Questionnaire** | Apseloff et al. 2013 | ✘ | ✘ | self | ✘ |
| 16 | **Questionnaire on Knowledge about Medical Research** | Asher et al. 2022 | ✔ | ✘ | self | ✘ |
| 17 | **Knowledge of Clinical Trials** | Campbell et al. 2008 | ✘ | ✔ | self and phone interview for follow up | 15-20 mins |
| 18 | **English Questionnaire (by J. Collins)** | Collins et al. 2023 | ✘ | ✘ | self | within a few minutes |
| 19 | **structured oral questionnaire** | Davis et al. 1998 | ✘ | ✔ | in-person interview | The entire interview was completed in about 25 minutes using the Southwestern Oncology Group (SWOG) form or 10–12 minutes using the Louisiana State University Medical Center— Shreveport (LSU) form |
| 20 | **Computer-based questionnaire** | De Oliveira et al. 2017 | ✘ | ✘ | self | ✘ |
| 21 | **Postconsent test** | Dresden et al. 2001 | ✘ | ✘ | self | ✘ |
| 22 | **10-item comprehension quiz** | Duvall Antonacopoulos et al. 2016 | ✔ | ✘ | self | ✘ |
| 23 | **A 20-item questionnaire.** | Eichner et al. 2020 | ✘ | ✔ | in person & telephone Interviews | ✘ |
| 24 | **questionnaire (A) and (B)** | Gammelgaard et al. 2004 | ✘ | ✘ | self | ✘ |
| 25 | **Questionnaire to assess patient comprehension** | Goldberger et al. 2011 | ✘ | ✘ | self | ✘ |
| 26 | **Three multiple-choice questions** | Griffin et al. 2006 | ✘ | ✘ | self | ✘ |
| 27 | **17 item true false questionnaire** | Harrison et al. 1995 | ✘ | ✘ | self | ✘ |
| 28 | **15-item placebo knowledge questionnaire** | Hughes et al. 2017 | ✘ | ✘ | self | 10 mins |
| 29 | **A structured questionnaire developed by the research team** | Jeong et al. 2012 | ✔ | ✘ | Questionnaire based interviews (patients); self (nurses) | ✘ |
| 30 | **Two survey questionnaires** | Kashur et al. 2023 | ✔ | ✘ | self | ✘ |
| 31 | **Nine-item questionnaire** | Krosin et al. 2006 | ✘ | ✘ | Oral administration. | ✘ |
| 32 | **Questionnaire developed by the authors** | Länsimies-Antikainen et al. 2010 | ✔ | ✔ | self | ✘ |
| 33 | **Questionnaire to assess participant perspective** | Mansour et al. 2015 | ✘ | ✘ | investigator | ✘ |
| 34 | **Questionnaire developed for this study** | Pope et al. 2003 | ✘ | ✘ | self | ✘ |
| 35 | **Questionnaire developed for the study** | Schmanski et al. 2021 | ✔ | ✘ | self | ✘ |
| 36 | **Multilingual survey developed by the authors** | Siao et al. 2014 | ✔ | ✘ | self | ✘ |
| 37 | **Questionnaire developed for the study** | Smith et al. 2016 | ✘ | ✘ | self | ✘ |
| 38 | **Questionnaire developed for the study** | Tadros et al. 2019 | ✘ | ✔ | self | ✘ |
| 39 | **Questionnaire developed for the study** | Verheggen et al. 1996 | ✔ | ✔ | questionnaire based interviews | ✘ |
| 40 | **Questionnaire developed for the study** | Weston et al. 1997 | ✘ | ✘ | self | ✘ |
| 41 | **standard examination** | Woodward et al. 1979 | ✘ | ✘ | self | ✘ |
| 42 | **the knowledge survey (8-item survey section)** | Hu et al. 2022 | ✔ | ✘ | self | Approximately 20 mins |
| 43 | **Knowledge test/ Patient consent quiz** | Norris et al. 1990 | ✘ | ✘ | self | ✘ |
| 44 | **The Electronic Informed Consent Attitudes Scale (eIC Attitudes Scale)** | Hu et al. 2022 | ✔ | ✔ | self | Approximately 20 mins |
| 45 | **Informed consent comprehension questionnaire** | Arora et al. 2011 | ✘ | ✔ | self | 15 - 20 minutes |
| 46 | **survey + indepth interview** | Ballard et al. 2020 | ✘ | ✘ | survey (self); Interviews (in-person or via telephone) | ✘ |
| 47 | **CRO-Biobank questionnaire for patients** | Cervo et al. 2013 | ✘ | ✘ | self | 15 mins |
| 48 | **enrollment quiz (Quiz1)** | Chaisson et al. 2011 | ✘ | ✘ | self | ✘ |
| 49 | **consent process questionnaire (CPQ)** | Criscione et al. 2003 | ✘ | ✘ | self | ✘ |
| 50 | **STRIPES Questionnaire** | Gertsman et al. 2020 | ✘ | ✘ | in person or by telephone | ✘ |
| 51 | **Questionnaire – Patient Understanding of Research** | Hutchison et al. 2007 | ✔ | ✔ | self | Range 5-20 minutes Patients: Mean 10.6 minutes Research nurses: Mean 8.8 minutes |
| 52 | **Belmont Beta Questionnaire** | Juan Salvadores et al. 2022 | ✔ | ✘ | self | ✘ |
| ✘ | **Consent Understanding Evaluation (CUE).** | Kass et al. 2015 | ✘ | ✘ | questionnaire based phone interview | 20 min |
| 54 | **post-test questionnaire;** | Koonrungsesomboon et al. 2017 | ✘ | ✘ | self | Median of 30 minutes (including time to read ICF and complete questionnaire) |
| 55 | **Informed Consent Evaluation Survey** | Lewis et al. 2015 | ✘ | ✘ | self | ✘ |
| 56 | **Closed-ended comprehension assessment (IC-C) Open-ended comprehension assessment (IC-O) Self-perception comprehension assessment (IC-SP)** | MacQueen et al. 2014 | ✘ | ✘ | self | ✘ |
| 57 | **Assessment of Consent recall Questionnaire (ACQ) Additional Questionnaire (AQ)** | Mboizi et al. 2017 | ✘ | ✘ | questionnaire based interview | ✘ |
| 58 | **informed consent assessment instrument (ICAI)** | Mexas et al. 2014 | ✘ | ✘ | interview | Administration of the ICAI was rapid Median time of 8 minutes (IQR 4-13 minutes) |
| 59 | **14 item questionnaire** | Miller et al. 1994 | ✘ | ✘ | telephone survey | ✘ |
| 60 | **Quality of consent evaluation questionnaire** | Minnies et al. 2008 | ✘ | ✘ | self (with assistance from study staff) | ✘ |
| 61 | **Postal Questionnaire** | Montgomery et al. 1998 | ✘ | ✘ | self | ✘ |
| 62 | **10-question test** | Ossemane et al. 2018 | ✘ | ✘ | interview | 10-15 min |
| 63 | **student comprehension tests and semi-structured interview** | Penn et al. 2010 | ✘ | ✘ | Verbally administered | ✘ |
| 64 | **not specified** | Ranjan et al. 2019 | ✘ | ✘ | self | ✘ |
| 65 | **30 item multiple-choice questionnaire** | Ravina et al. 2010 | ✔ | ✘ | self | ✘ |
| 66 | **38-item telephone survey** | Roth et al. 2021 | ✔ | ✘ | telephone survey | ✘ |
| 67 | **Questionnaire (not named)** | Russel et al. 2005 | ✘ | ✔ | Verbally by researcher | ✘ |
| 68 | **12-question survey** | Shamy et al. 2019 | ✘ | ✘ | Surveys (in person or over the telephone) | ✘ |
| 69 | **13-item posttest instrument** | Shelton et al. 2015 | ✔ | ✔ | self | Not explicitly stated, but total visit time did not exceed 30 minutes |
| 70 | **Consent Understanding Evaluation—Refined (CUE-R)** | Taylor et al. 2021 | ✘ | ✘ | questionnaire based phone interview | 15-20 minutes |
| 71 | **Eight-item true/false/don't know questionnaire about ddI** | Tindall et al. 1994 | ✘ | ✘ | self | ✘ |
| 72 | **Informed Decision-making (IDM) questionnaire** | Van Den Bergh et al. 2009 | ✘ | ✔ | self | ✘ |
| 73 | **Questionnaire** | Van Stuijvenberg et al. 1998 | ✘ | ✘ | self | ✘ |
| 74 | **Participatory and Informed Consent (PIC) measure** | Wada et al. 2017 | ✔ | ✔ | researcher analysed the audio-recordings | Mean of 56 minutes in phase 2 evaluation |
| 75 | **audio-recordings of information appointments** | Wade et al. 2009 | ✘ | ✔ | researcher analysed the audio-recordings | ✘ |
| 76 | **Informed Consent Survey** | Wirshing et al. 1998 | ✘ | ✘ | verbally via study coordinator | ✘ |
| 77 | **questionnaire** | Young-Afat 2021 | ✘ | ✘ | self | ✘ |
| 78 | **the patient questionnaire** | Yuval et al. 2000 | ✘ | ✘ | self | ✘ |
| 79 | **10-item questionnaire** | Zhang et al. 2017 | ✘ | ✘ | self | **Video Group:** 5.6 ± 5.4min **Control group**: 12.3 ± 6.7min |
| 80 | **(1) Clinical trial knowledge sclae adapted form of a 7-item scale about trial methods and rationale for them ;; (3) se-designed 13-item scale to assess understanding of the trial (IBIS-II DCIS trial understanding scale),** | Juraskova et al. 2008 | ✔ | ✔ | Self (survey) and interviews (phone). | ✘ |
| 81 | **MacArthur Competency Assessment Tool for Clinical Research (MacCAT-CR). (The interviewer adapted the MacCAT-CR questions)** | Karlawish et al. 2002 | ✔ | ✔ | interview | ✘ |
| 82 | **MacArthur Competence Assessment Tool for Clinical Research (MacCAT-CR), adapted for the LD setting as MacLiver** | Freeman et al. 2013 | ✔ | ✔ | computerized administration | ✘ |
| 83 | **Quality of Informed Consent (QuIC) scale - Subjective Understanding subscale; 6 statements developed by the research team that reflected factual information present in all 3 e-consent versions; Decision-Making Control Instrument** | Golembiewsky et al. 2021 | ✔ | ✔ | tablet-based, self-guided | ✘ |
| 84 | **the Understanding Treatment Disclosures Scale (UTD)** | Ghormley et al. 2011 | ✔ | ✔ | verbally by examiner. | 20 mins |
| 85 | **perceived understanding, seven mirroring questions (7Q-PAT); and Patient Understanding of Research (Q-PUR) questionnaire** | Dellson et al. 2019 | ✔ | ✔ | self | ✘ |
| 86 | **Process-Quality of Informed Consent (P-QIC)** | Cohn 2009 | ✔ | ✔ | The tool was administered through observation of scripted video vignettes using volunteer actors to depict informed consent encounters; researchers completed the observational tool while viewing these standardised video scenarios. | ✘ |
| 87 | **informed consent assessment tool (ICAT)** | Chapman et al. 2015 | ✔ | ✘ | verbally by interviewers. | ✘ |
| 88 | **half-standardised interview guideline** | Behrendt et al. 2011 | ✘ | ✔ | interview | 18 to 53 min |
| 89 | **subject understanding interviews.** | Benson et al. 1985 | ✔ | ✔ | interview | Depression study: Mean disclosure length was 9 minutes. Schizophrenia study: Mean disclosure length was 41 minutes. |
| 90 | **Framework used for the interview** | Beranger et al. 2019 | ✔ | ✔ | interview | ✘ |
| 91 | **interview addressing the level of understanding** | Chappuy et al 2010 | ✘ | ✔ | interview | mean for first interviews = 52.3 minutes (15–120 min; SD 19.8) mean for second interviews = 39.4 minutes (15–75 min; SD 16.6) |
| 92 | **Interview Guide: English** | Choi et al 2019 | ✘ | ✘ | interview | 40 minutes to 1 hour 15 minutes, with a median of 45 minutes (average 48.5 minutes). |
| 93 | **Questions to construct the vaccine knowledge score** | da Fonseca et al. 1999 | ✘ | ✘ | interview | ✘ |
| 94 | **Semi-structured interview guide** | Das et al. 2014 | ✘ | ✘ | interview | average 30 minutes |
| 95 | **A semi-structured questionnaire consisting of 32 questions.** | Diemert et al. 2017 | ✘ | ✔ | questionnaire based interview | 10 mins |
| 96 | **Interview grid (ad hoc for study)** | Falvo et al. 2021 | ✘ | ✘ | interview | one hour |
| 97 | **interview forms: (a) for early contact (b) for recal** | Fortney et al. 1999 | ✘ | ✘ | interview | ✘ |
| 98 | **Recall of trial details** | Hofmeijer et al. 2007 | ✘ | ✘ | Telephone interview. | ✘ |
| 99 | **interview guide** | Howard et al. 1981 | ✘ | ✘ | interview | ✘ |
| 100 | **structured questionnaire** | Kass et al. 2009 | ✘ | ✘ | In-person or by telephone interview | 30–40 minutes |
| 101 | **Semi-structured interview** | Leach et al. 1999 | ✘ | ✘ | interview | ✘ |
| 102 | **Focus group discussion guide with 8 questions** | Meneguin et al. 2014 | ✘ | ✘ | focus group discussion | Approximately two hours |
| 103 | **Patient Interview Questions** | Miller et al. 2013 | ✘ | ✘ | questionnaire based interview | ✘ |
| 104 | **patient interview guide** | Mills et al. 2003 | ✘ | ✘ | interview | 45 and 105 minutes (average 60 minutes) |
| 105 | **Semi-structured questionnaire** | Moodley et al. 2005 | ✘ | ✘ | interview | Approx. 20 minutes |
| 106 | **in-depth interview guide (for healthcare workers) in-depth interview guide (for patients) Observation guides** | Nguyen et al. 2023 | ✘ | ✘ | interview | ✘ |
| 107 | **Semi-structured interview guide** | Rose et al. 2013 | ✘ | ✘ | interview | ✘ |
| 108 | **Semi-structured interview guide designed for this study** | Sand et al. 2008 | ✘ | ✘ | interview | 6 to 42 minutes (median 12 minutes) |
| 109 | **Semi-structured qualitative interview** | Searight et al. 1996 | ✘ | ✘ | interview | ✘ |
| 110 | **Ad hoc questionnaire specifically devised for this survey** | Sanchini et al. 2014 | ✘ | ✘ | interview | ✘ |
| 111 | **Structured questionnaire** | Sarkar et al. 2010 | ✘ | ✘ | interview | ✘ |
| 112 | **Standardized questionnaire** | Schats et al. 2003 | ✘ | ✘ | interview | ✘ |
| 113 | **Roter Interactional Analysis System (RIAS)** | Hlubocky et al. 2008 | Not Applicable | ✔ | interview | ✘ |
